# Supplementary material for: Reinforced optical cage systems enable drift-free single-molecule localization microscopy
Source: Commun Eng. 2025 Dec 15;5:13. doi: 10.1038/s44172-025-00566-4 (PMC12820167; doi:10.1038/s44172-025-00566-4)
Supplement: Supplementary file 2 — Supplementary Information [file 44172_2025_566_MOESM2_ESM.pdf]

Supplementary Information

Reinforced optical cage systems enable drift-free single-molecule localization microscopy

Hao Qiu[1,2,‡], Matthew C. Tang[1,3,‡], Selene K. Roberts[1], Guoli Li[4], Rong Su[4], Marisa L. Martin-Fernandez[1], David T. Clarke[1], Shugang Liu[5], Xiaojie Liu[2] & Lin Wang[1*]

1. Central Laser Facility, Research Complex at Harwell, Rutherford Appleton Laboratory, Science and Technology Facilities Council, Didcot OX11 0QX, UK
2. School of Electrical and Information Engineering, Jiangsu University of Technology, Changzhou, Jiangsu 213001, China
3. Division of Structural Biology, Nuffield Department of Medicine, University of Oxford, Oxford OX3 7BN, UK
4. Shanghai Institute of Optics and Fine Mechanics, Chinese Academy of Sciences, Shanghai 201800, China
5. RayCage (Zhenjiang) Photoelectric Technology Co., Ltd, Zhenjiang, Jiangsu 212004, China

‡ These authors contributed equally

* Corresponding author: lin.wang@stfc.ac.uk

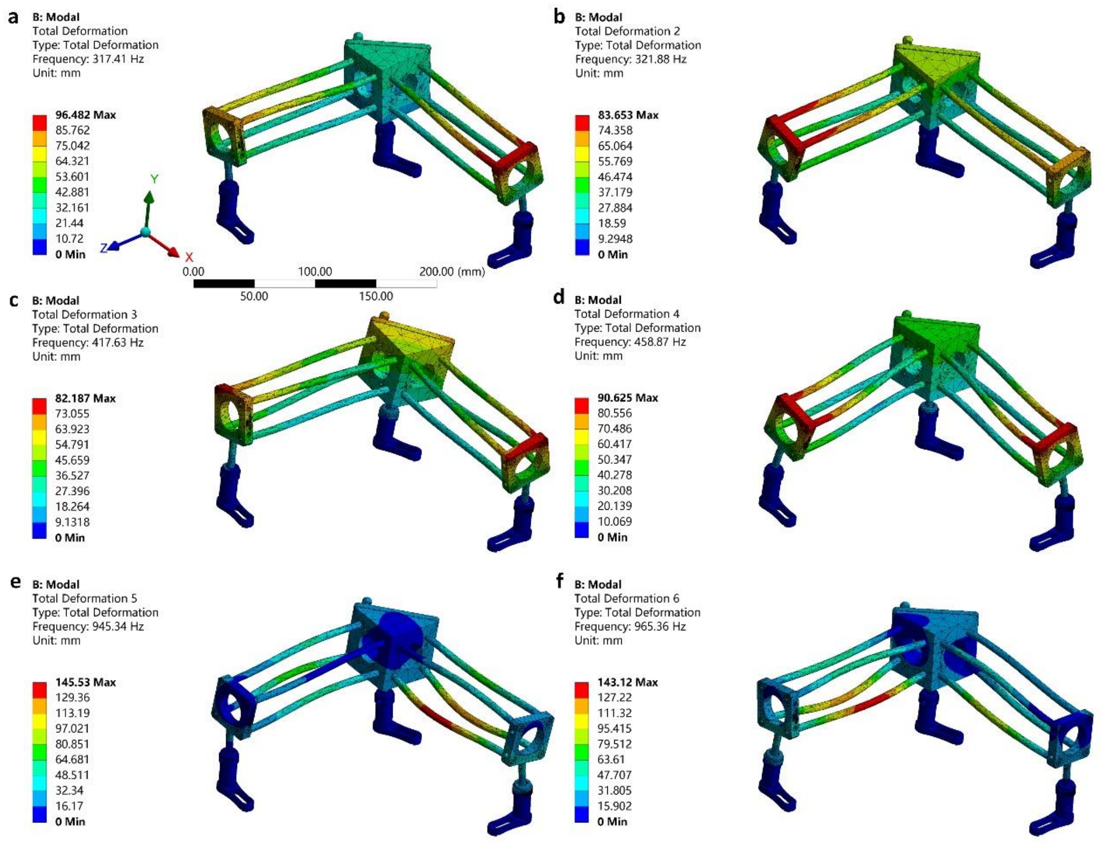

Figure S1. Vibration modal analysis of the right-angle kinematic mirror mount assembly using conventional optical cage systems. (**a**-**f**) The first six modes of the natural frequencies and corresponding mode shapes of the assembly.

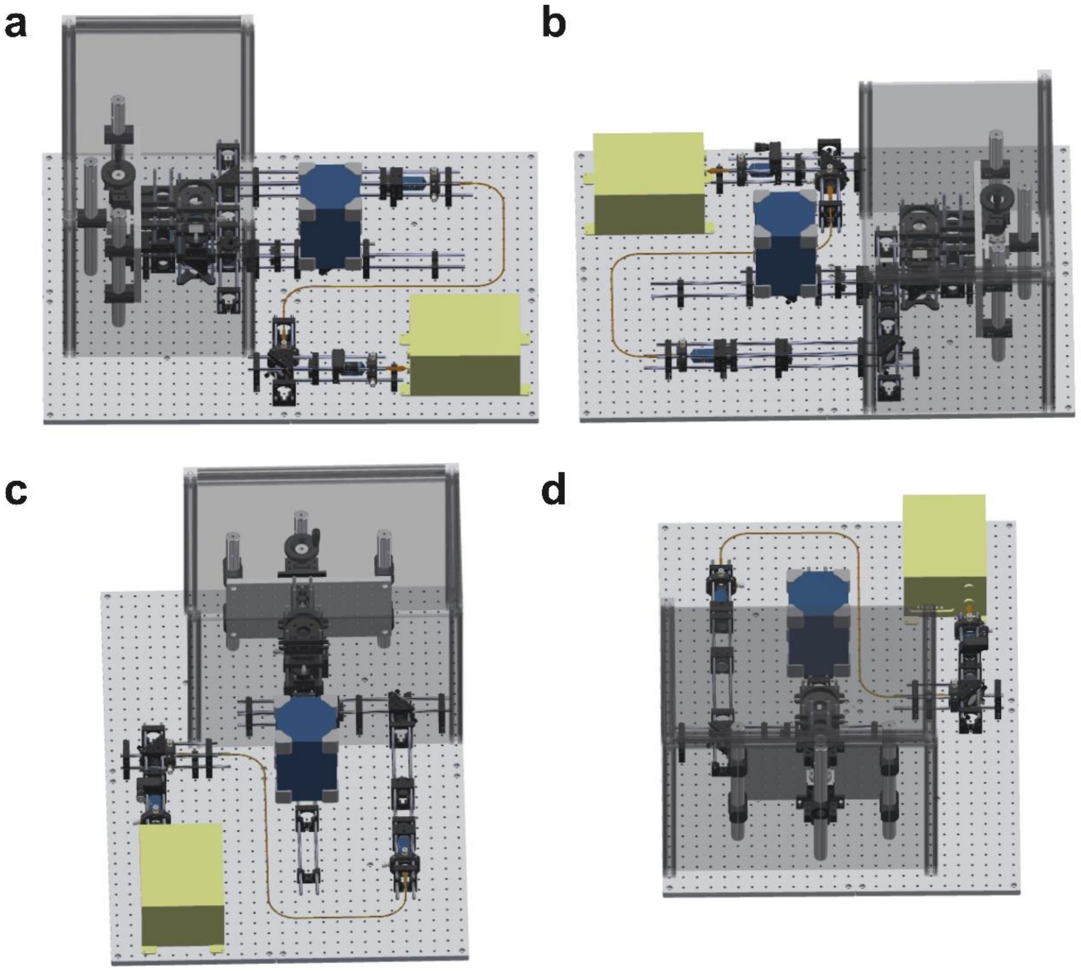

Figure S2. Isometric projection of the three-dimensional ROCS microscope design. (**a**-**d**) Views from the front, back, left, and right sides.

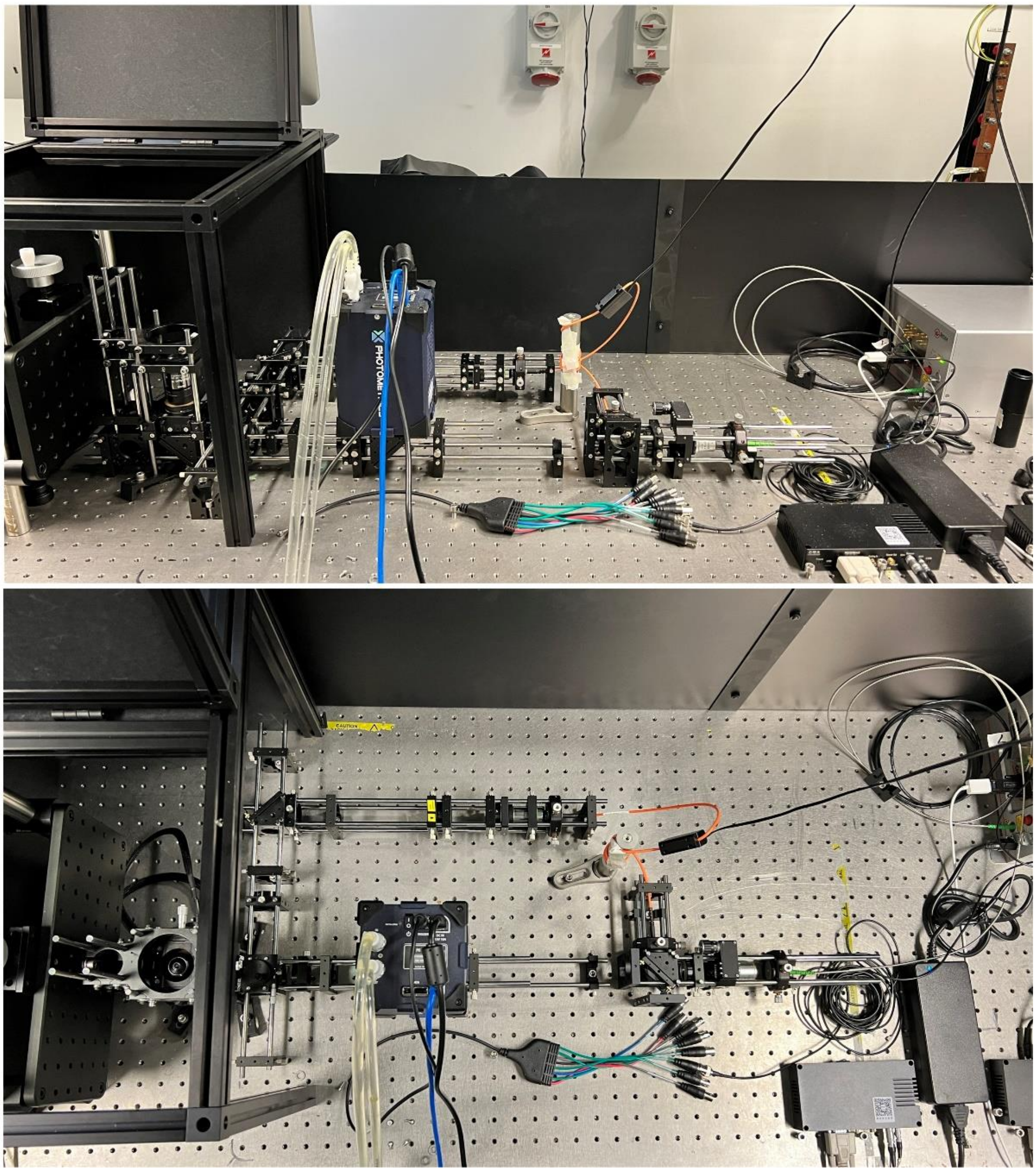

Figure S3. ROCS microscope photos. **a** Front view perspective of the system. **b** Top view perspective of the system.

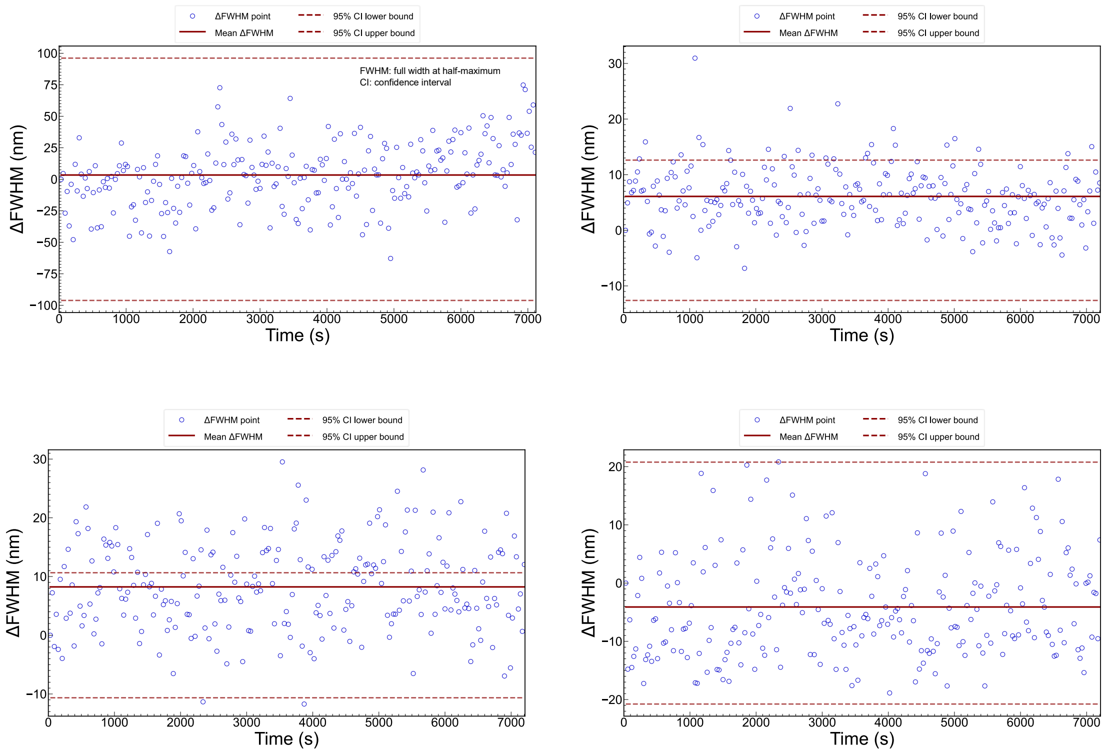

Fig S4. Axial drift evaluation in conventional widefield fluorescence microscopy using the ROCS microscope for all datasets, except for the one shown in Fig. 4**b**. The scatter plot shows the change in PSF size averaged across all beads in a frame. The solid red line represents the mean change in PSF size across all frames while the dashed red lines represent the bounds of the 95% confidence interval. The mean change in the PSF FWHM was 3.4 ± 25.2 nm, 6.1 ± 5.2 nm, 8.2 ± 7.4 nm and -4.1 nm ± 8.6 nm, respectively (the panels are arranged in the order: top-left, top-right, bottom-left, bottom-right).

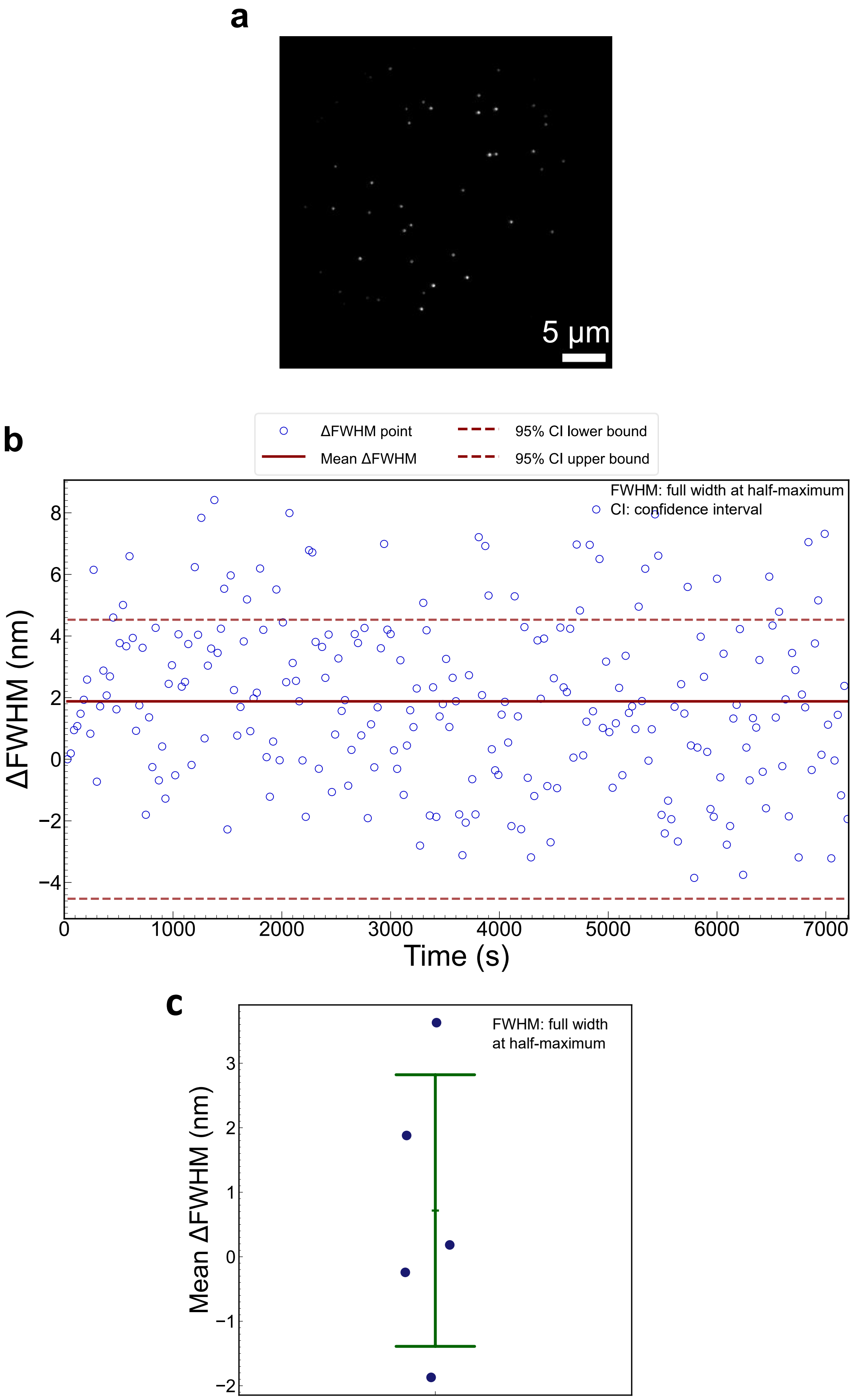
a
5 µm
b
ΔFWHM point
Mean ΔFWHM
95% CI lower bound
95% CI upper bound
FWHM: full width at half-maximum
CI: confidence interval
ΔFWHM (nm)
Time (s)
c
FWHM: full width at half-maximum
Mean ΔFWHM (nm)

Fig. S5 Axial drift evaluation in conventional widefield fluorescence microscopy using the ROCS microscope with autofocus. **a** A representative widefield image of the fluorescent beads. **b** Change in PSF size of the fluorescent beads over 2 hours from a representative image series, as measured by the change in the FWHM of the Gaussian fit. The mean change in the PSF FWHM was 1.9 ± 2.7 nm. The scatter plot shows the change in PSF size averaged across all beads in a frame. The solid red line represents the mean change in PSF size across all frames while the dashed red lines represent the bounds of the 95% confidence interval. **c** Dot plot of mean changes in the PSF FWHM of all five experimental repeats, showing an overall mean change of 0.7 ± 2.1 nm. The dots represent the mean change from each experimental iteration while the central bar represents the overall mean change. The upper and lower bars denote one standard deviation.

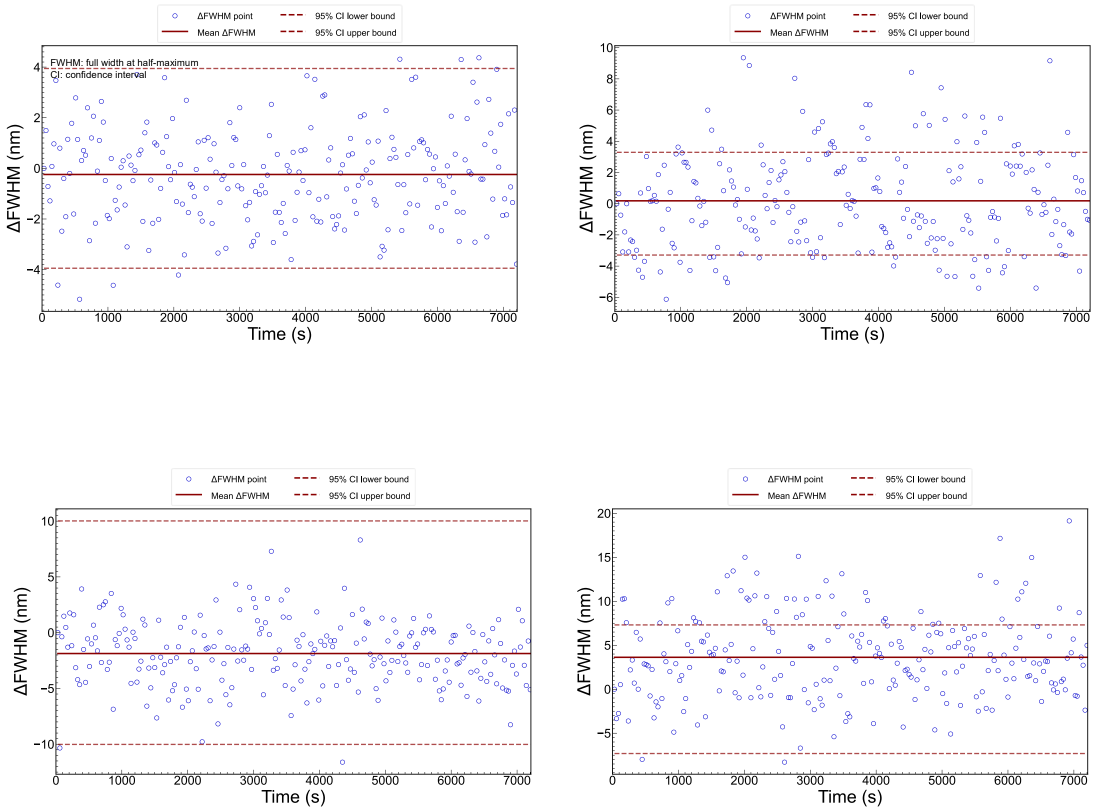

Fig S6. Axial drift evaluation in conventional widefield fluorescence microscopy using the ROCS with autofocus for all datasets, except for the one shown in Fig. S5**b**. The scatter plot shows the change in PSF size averaged across all beads in a frame. The solid red line represents the mean change in PSF size across all frames while the dashed red lines represent the bounds of the 95% confidence interval. The mean change in the PSF FWHM was -0.2 ± 1.8 nm, 0.2 ± 3.1 nm, -1.9 ± 2.9 nm and 3.6 nm ± 4.8 nm, respectively (from the top-left panel clockwise).

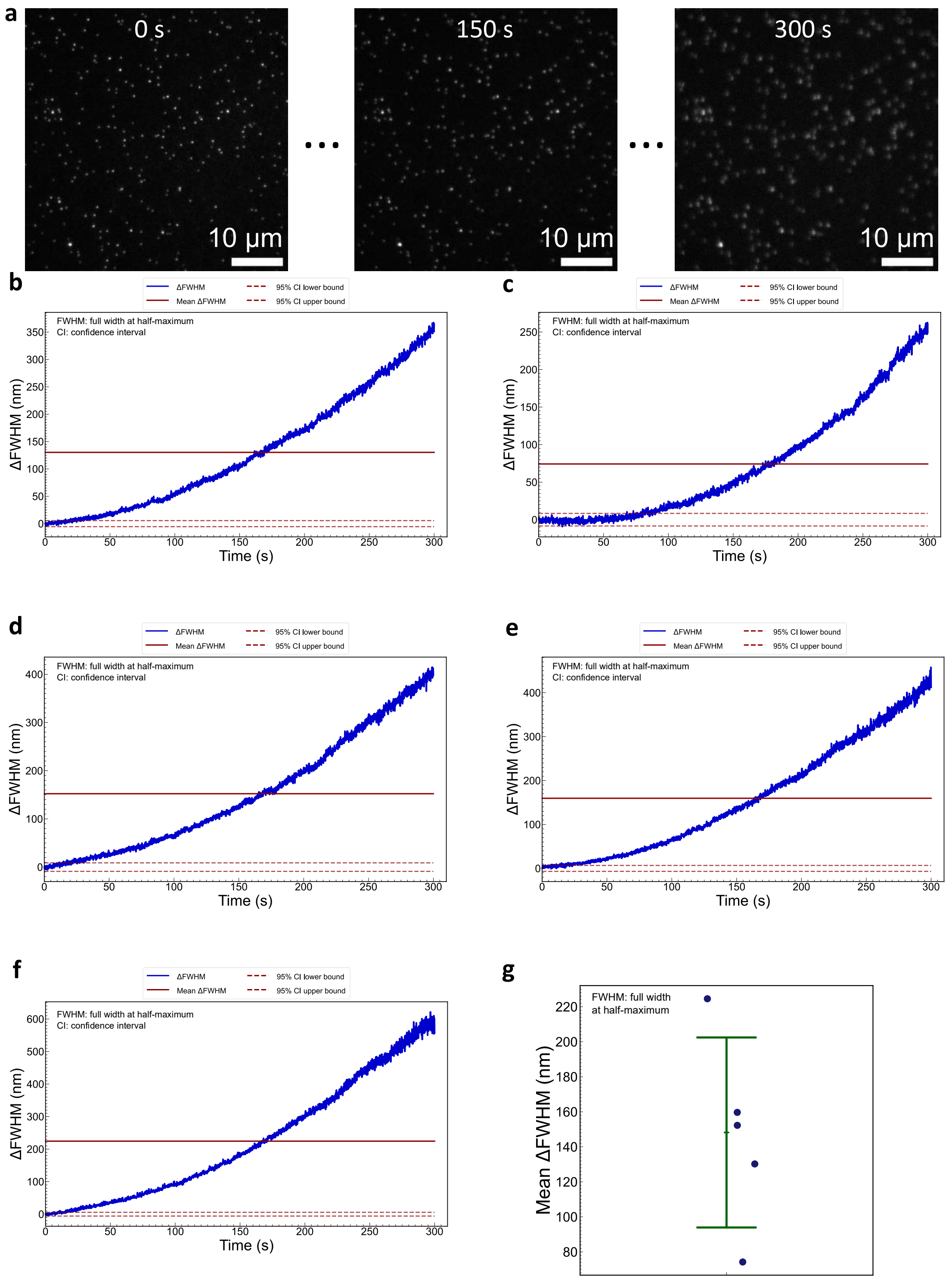

Figure S7. Axial drift evaluation in conventional widefield fluorescence microscopy using an off-the-shelf microscope without any active drift stabilization. **a** Representative time-lapse images of fluorescent beads acquired over 5 minutes. Progressive changes in the PSF size of the fluorescent beads indicate axial drift. (**b**-**f**) The scatter plot shows the change in PSF size averaged across all beads in a frame. The solid red line represents the mean change in PSF size across all frames while the dashed red lines represent the bounds of the 95% confidence interval. The mean change in the PSF FWHM was (from **b** to **f**) 130.2 ± 105.4 nm, 74.2 ± 76.3 nm, 152.3 ± 119.7 nm, 159.7 ± 127.8 nm and 224.6 ± 180.7 nm, respectively. **g** Dot plot of mean changes in the PSF FWHM of all five experimental

repeats, showing an overall mean change of 148.3 ± 54.3 nm. The dots represent the mean change from each experimental iteration while the central bar represents the overall mean change. The upper and lower bars denote one standard deviation.

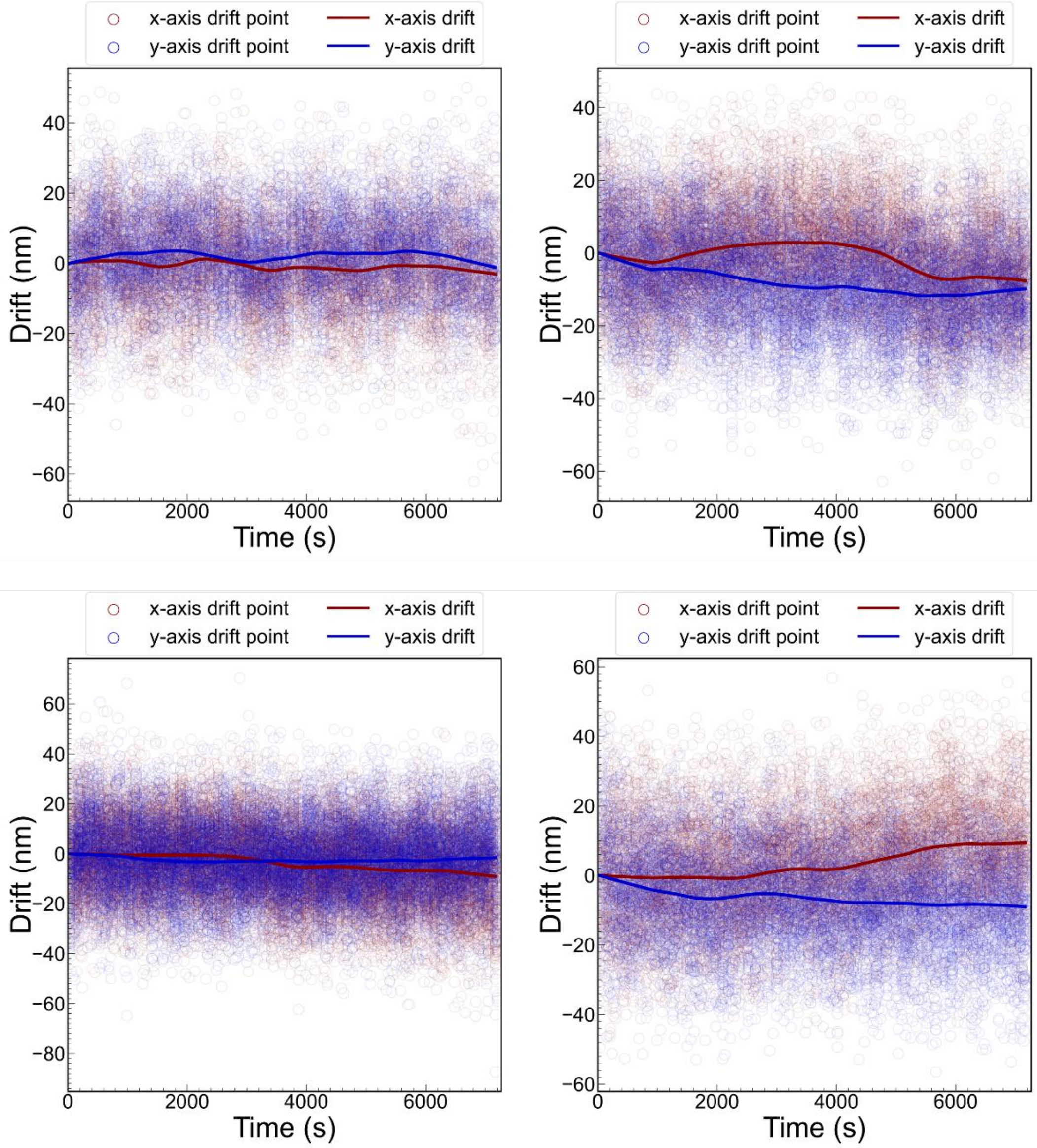

Figure S8. Drift trajectories of the fluorescent beads over 2 hours from all image series, except the one shown in Fig.5**a**, acquired using the ROCS microscope (the panels are arranged in the order: top-left, top-right, bottom-left, bottom-right).

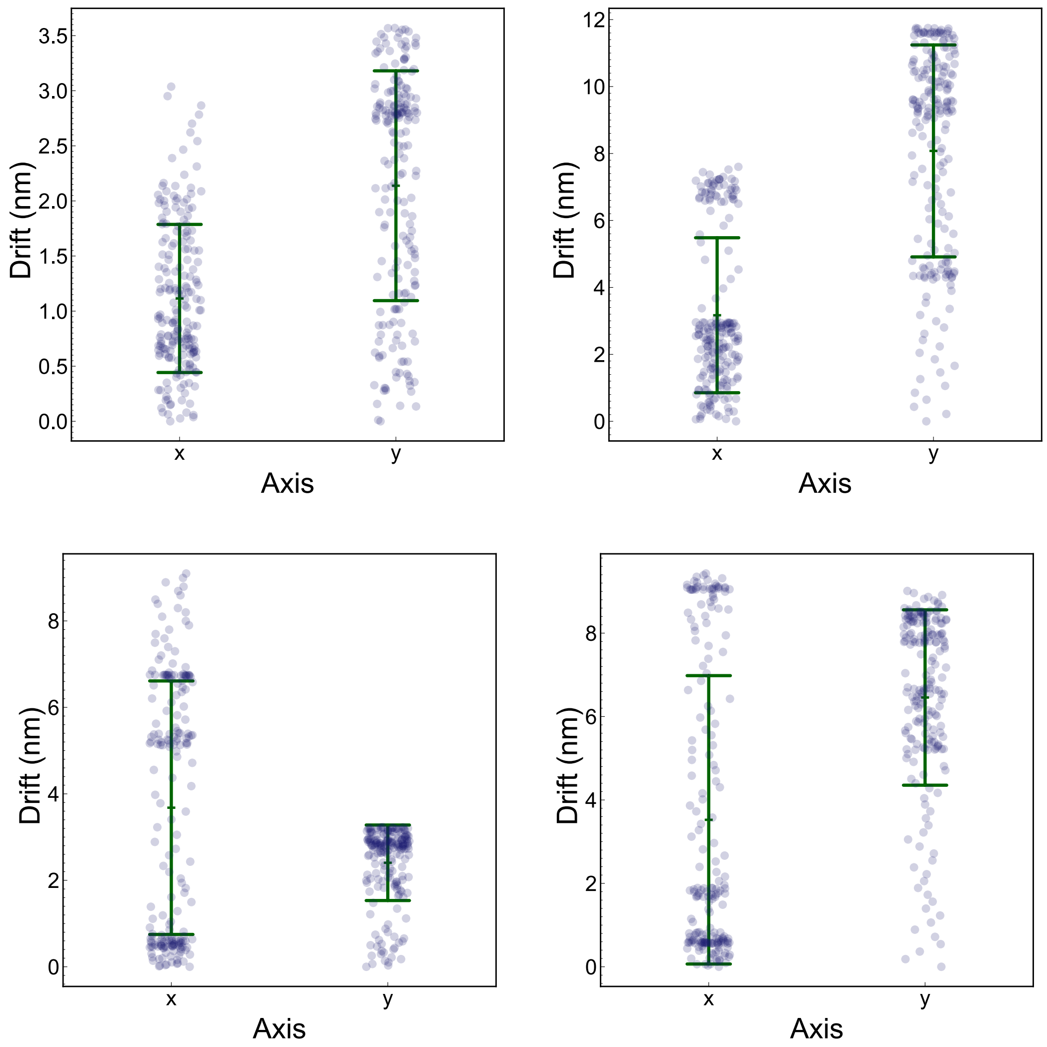

Figure S9. Dot plots of the absolute values of the drift along the x-axis and y-axis for the trajectories plotted in Figure S8. The dots represent the drift trajectory points, the central bar represents the mean drift, and the upper and lower bars represent one standard deviation. The mean drift was 1.1 ± 0.7 nm and 2.1 ± 1.0 nm, 3.2 ± 2.3 nm and 8.1 ± 3.2 nm, 3.7 ± 2.9 nm and 2.4 ± 0.9 nm, 3.5 ± 3.4 nm and 6.5 ± 2.1 nm, along the x-axis and y-axis, respectively (the panels are arranged in the order: top-left, top-right, bottom-left, bottom-right).

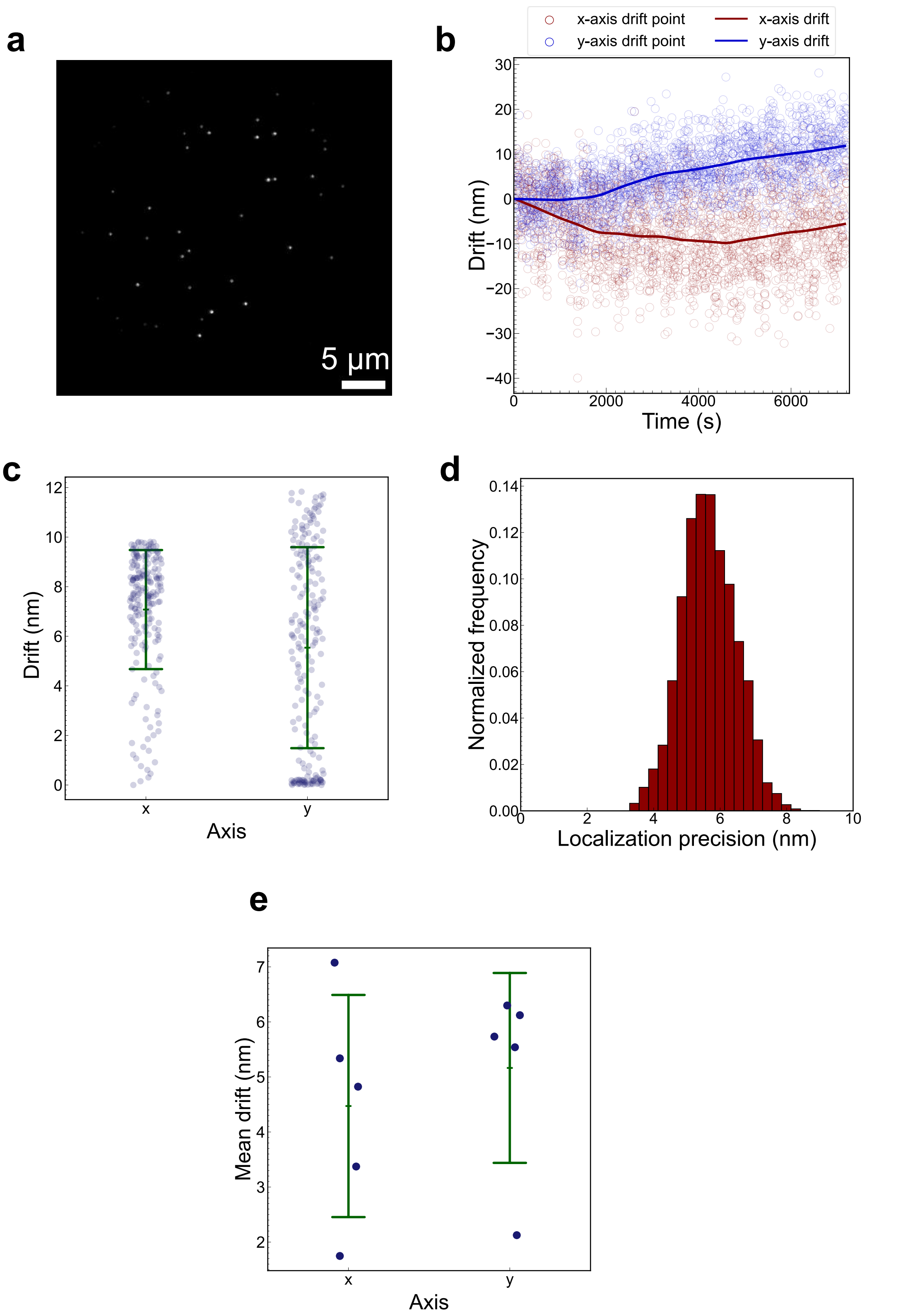

Figure S10. Lateral drift quantification in conventional widefield fluorescence microscopy using the ROCS microscope with autofocus. **a** A representative widefield image of the fluorescent beads. **b** Drift trajectory of the fluorescent beads over 2 hours from the representative image series. The scatter plot shows the positions of the beads which were localized using a Gaussian fit in ThunderSTORM while the curves represent the drift trajectories composed of the average drift of all beads as calculated from fiducial marker tracking. **c** Dot plot of the absolute

values of the drift along the x-axis and y-axis for the trajectory plotted in **b**. The dots represent the drift trajectory points, the central bar represents the mean drift, and the upper and lower bars represent one standard deviation. The mean drift was 7.1 ± 2.4 nm and 5.5 ± 4.0 nm along the x-axis and y-axis respectively. **d** Localization precision histogram of fluorescent beads in the image series plotted in **b**. The mean localization precision was 5.7 ± 0.8 nm. **e** Dot plot of the mean drifts from 5 experimental iterations. The mean drift from all iterations was 4.5 ± 2.0 nm along the x-axis and 5.2 ± 1.7 nm along the y-axis. The dots represent the mean drifts from each experimental iteration while the central bar represents the overall mean drift. The upper and lower bars denote one standard deviation.

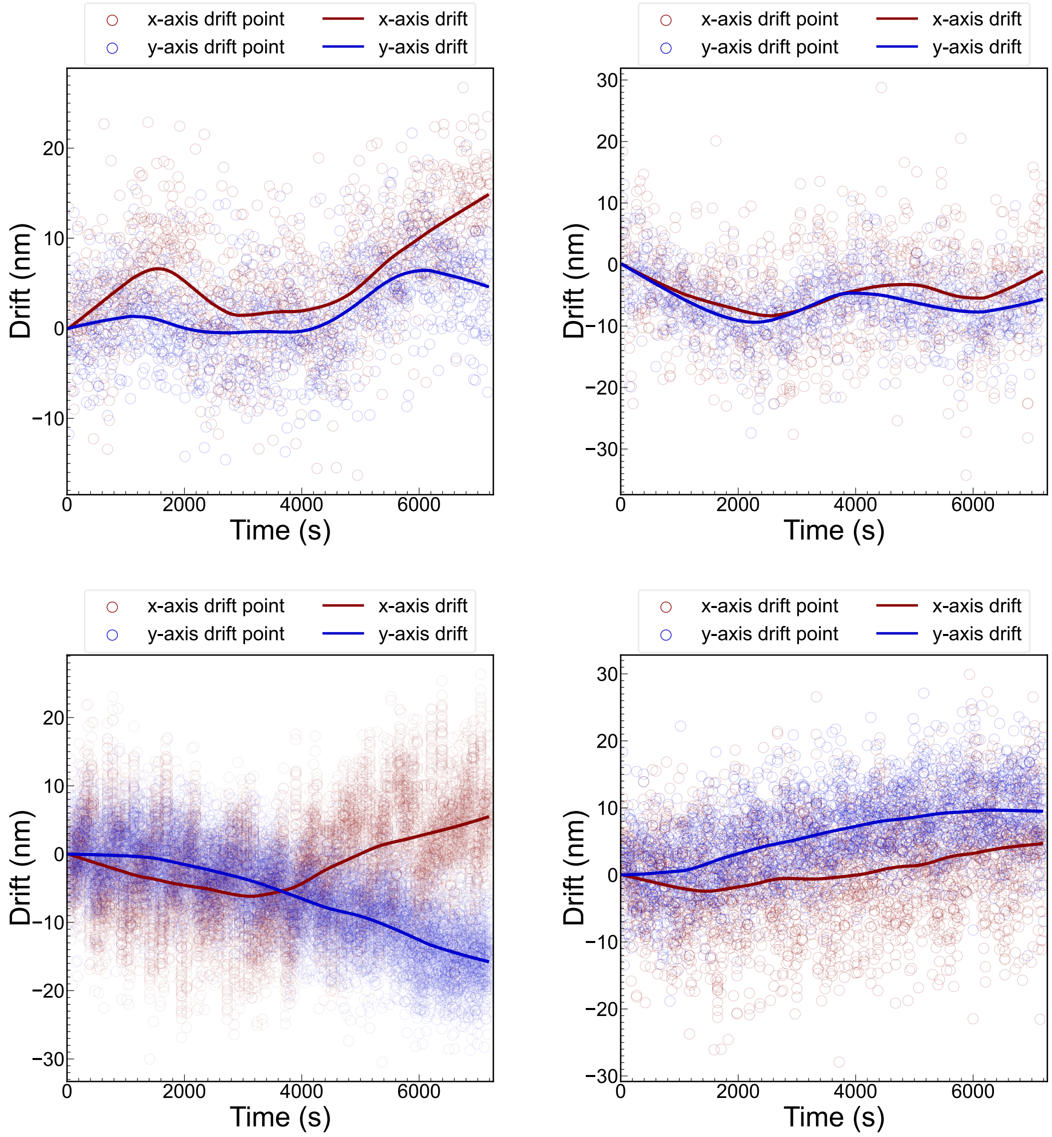

Figure S11. Drift trajectories of the fluorescent beads over 2 hours from all image series, except the one shown in Fig. S10**b**, acquired using the ROCS microscope with autofocus. The scatter plot shows the positions of the beads which were localized using a Gaussian fit in ThunderSTORM while the curves represent the drift trajectories composed of the average drift of all beads as calculated from fiducial marker tracking (the panels are arranged in the order: top-left, top-right, bottom-left, bottom-right).

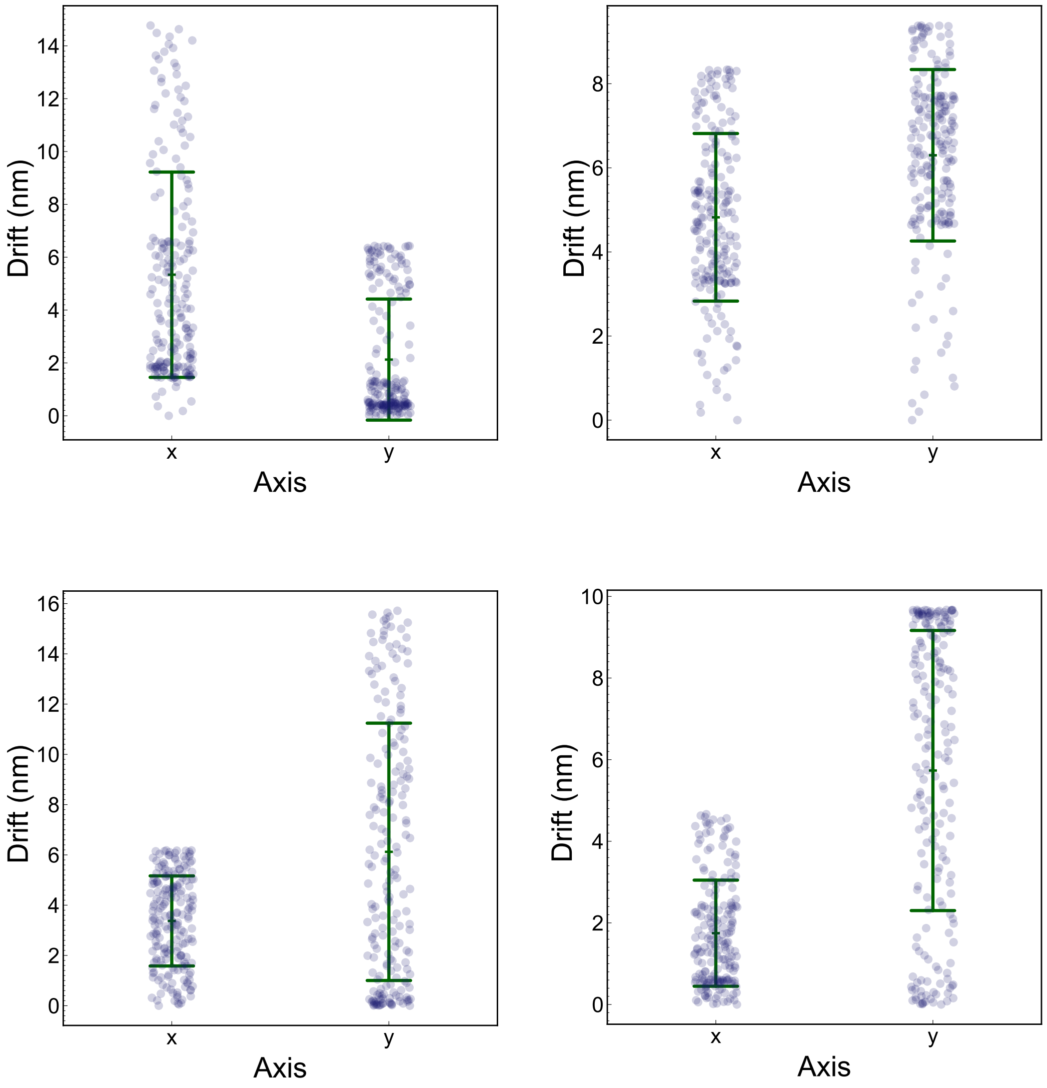

Figure S12. Dot plots of the absolute values of the drift along the x-axis and y-axis for the trajectories plotted in Figure S11. The dots represent the drift trajectory points, the central bar represents the mean drift, and the upper and lower bars represent one standard deviation. The mean drift was 5.3 ± 3.9 nm and 2.1 ± 2.3 nm, 4.8 ± 2.0 nm and 6.3 ± 2.0 nm, 3.4 ± 1.8 nm and 6.1 ± 5.1 nm, 1.7 ± 1.3 nm and 5.7 ± 3.4 nm, along the x-axis and y-axis, respectively (the panels are arranged in the order: top-left, top-right, bottom-left, bottom-right).

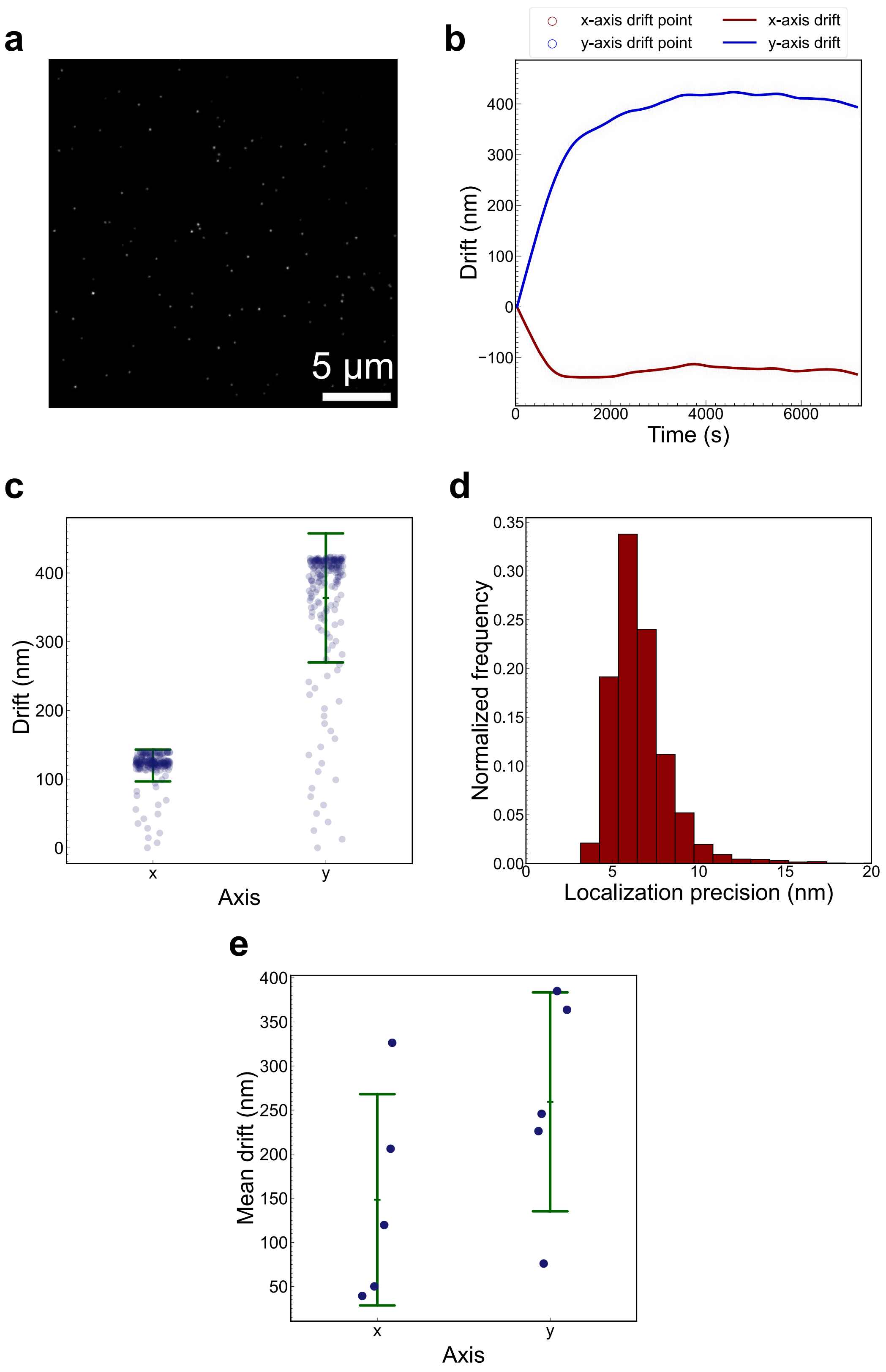

Figure S13. Lateral drift quantification in conventional widefield fluorescence microscopy using an off-the-shelf microscope with active focal-plane stabilization. **a** A representative widefield image of the fluorescent beads. **b** Drift trajectory of the fluorescent beads over 2 hours from the representative image series. The scatter plot shows the positions of the beads which were localized using a Gaussian fit in ThunderSTORM while the curves represent the drift trajectories composed of the average drift of all beads as calculated from fiducial marker tracking. **c** Dot

plot of the absolute values of the drift along the x-axis and y-axis for the trajectory plotted in **b**. The dots represent the drift trajectory points, the central bar represents the mean drift, and the upper and lower bars represent one standard deviation. The mean drift was 119.7 ± 23.1 nm and 363.8 ± 93.7 nm along the x-axis and y-axis respectively. **d** Localization precision histogram of fluorescent beads in the image series plotted in **b**. The mean localization precision was 6.4 ± 2.3 nm. **e** Dot plot of the mean drifts from 5 experimental iterations. The mean drift from all iterations was 148.3 ± 119.8 nm along the x-axis and 259.4 ± 124.1 nm along the y-axis. The dots represent the mean drifts from each experimental iteration while the central bar represents the overall mean drift. The upper and lower bars denote one standard deviation.

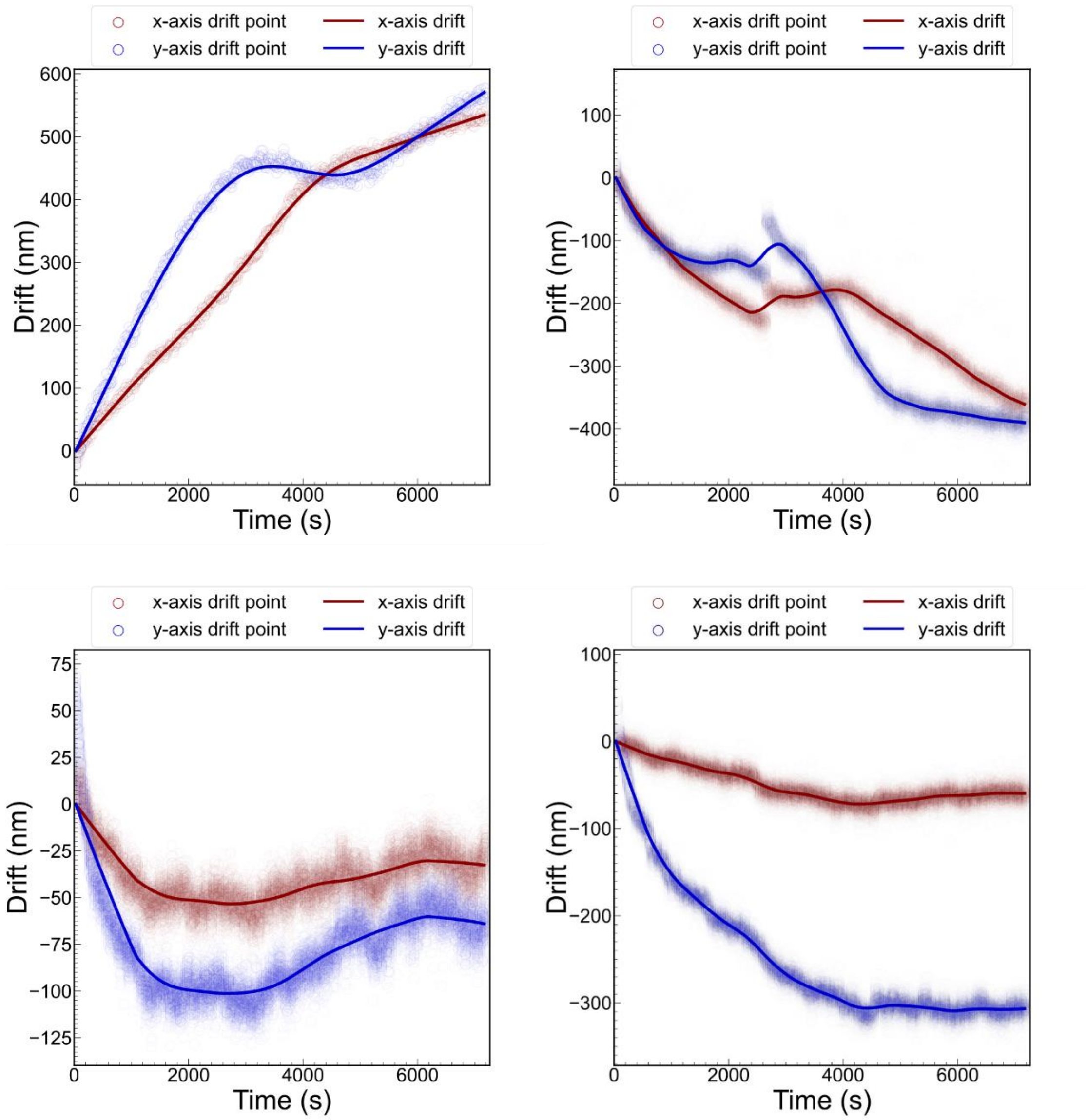

Figure S14. Drift trajectories of the fluorescent beads over 2 hours from all image series, except the one shown in Fig. S13**b**, acquired using an off-the-shelf microscope with active focal-plane stabilization. The scatter plot shows the positions of the beads which were localized using a Gaussian fit in ThunderSTORM while the curves represent the drift trajectories composed of the average drift of all beads as calculated from fiducial marker tracking (the panels are arranged in the order: top-left, top-right, bottom-left, bottom-right).

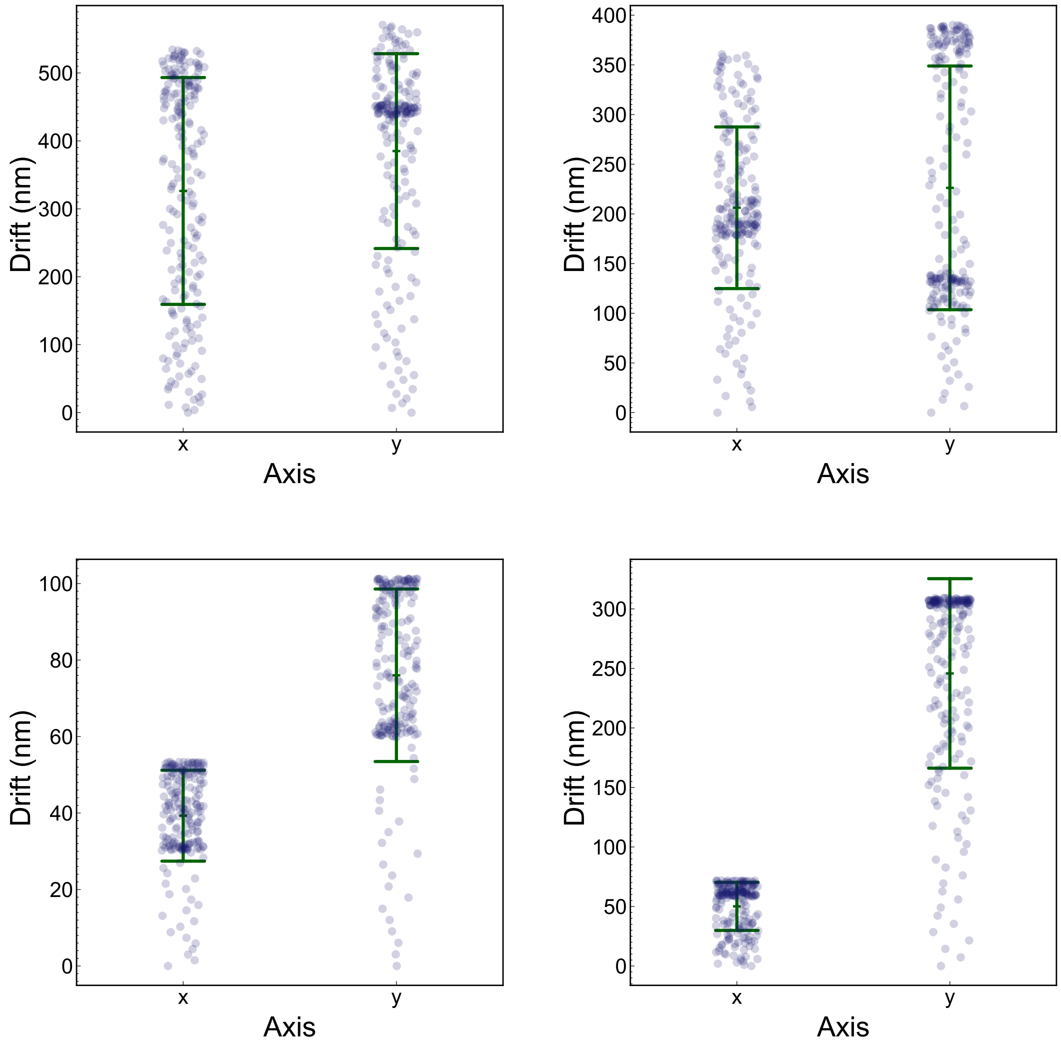

Figure S15. Dot plots of the absolute values of the drift along the x-axis and y-axis for the trajectories plotted in Figure S14. The dots represent the drift trajectory points, the central bar represents the mean drift, and the upper and lower bars represent one standard deviation. The mean drift was 326.3 ± 166.5 nm and 385.0 ± 143.1 nm, 206.2 ± 81.1 nm and 226.2 ± 122.3 nm, 39.3 ± 11.9 nm and 76.0 ± 22.5 nm, 50.1 ± 20.2 nm and 245.8 ± 79.4 nm, along the x-axis and y-axis, respectively (the panels are arranged in the order: top-left, top-right, bottom-left, bottom-right).

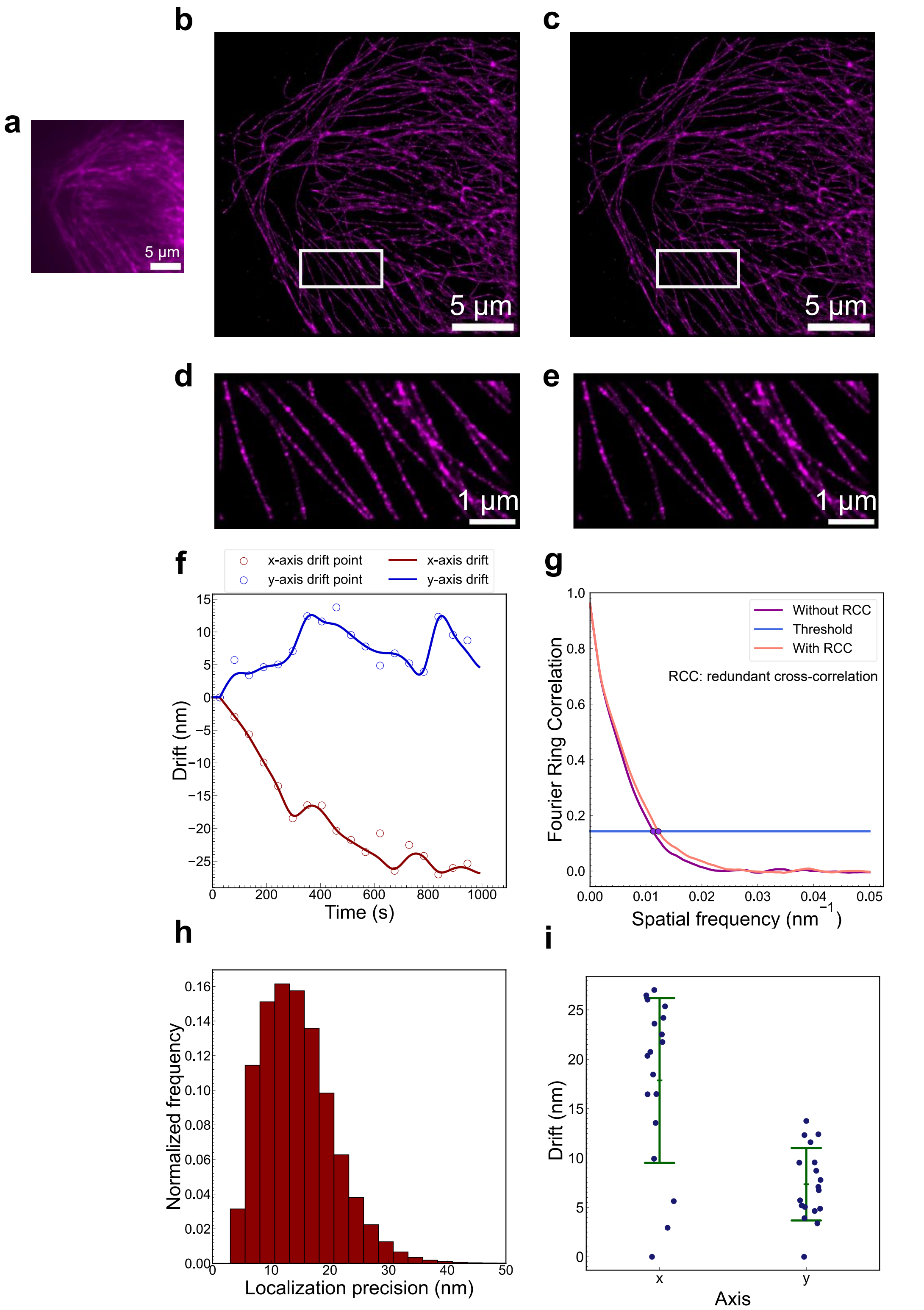

Figure S16. The second iteration of STORM imaging of α-Tubulin-AF647 in the microtubule network in fixed HeLa cells using the ROCS-STORM system. **a** Widefield image. **b** STORM image without drift correction and **c** drift-corrected STORM image using redundant cross-correlation (RCC). **d** and **e** Magnified view of the boxed area in **b** and **c**. **f** Drift trajectory of the image series from STORM over about 15 minutes (33000 frames). The trajectory was calculated using RCC with each circle representing the drift of one bin, i.e. the drift of one image subset relative to

the first subset. A total of 18 bins were used. **g** Fourier ring correlation (FRC) resolution evaluation of the STORM images with (**c**) and without (**b**) drift correction, demonstrating 88.6 nm and 81.9 nm resolution before and after drift correction, respectively. A threshold of 0.143 was used. **h** Localization precision histogram of the image shown in **b**. The mean localization precision was 14.5 ± 6.1 nm. **i** Dot plot of the absolute values of drift from **f**. The mean drift was 17.9 ± 8.1 nm along the x-axis and 7.3 ± 3.6 nm along the y-axis. The dots represent the drift data points while the central bar represents the mean drift. The upper and lower bars denote one standard deviation.

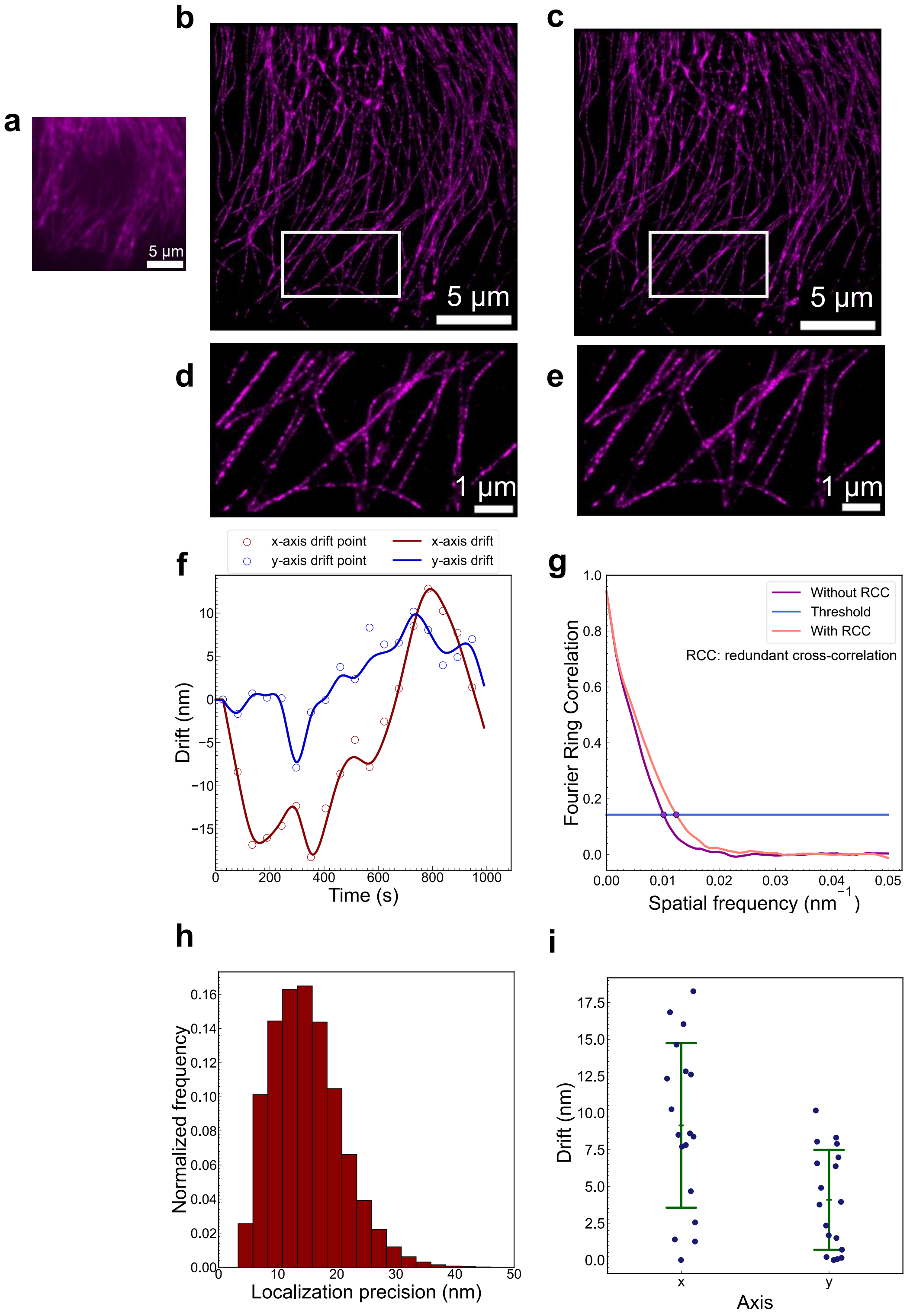

Figure S17. The third iteration of STORM imaging of α-Tubulin-AF647 in the microtubule network in fixed HeLa cells

using the ROCS-STORM system. **a** Widefield image. **b** STORM image without drift correction and **c** drift-corrected STORM image using redundant cross-correlation (RCC). **d** and **e** Magnified view of the boxed area in **b** and **c**. **f** Drift trajectory of the image series from STORM over about 15 minutes (33000 frames). The trajectory was calculated using RCC with each circle representing the drift of one bin, i.e. the drift of one image subset relative to the first subset. A total of 18 bins were used. **g** Fourier ring correlation (FRC) resolution evaluation of the STORM images with (**c**) and without (**b**) drift correction, demonstrating 98.7 nm and 80.7 nm resolution before and after drift correction, respectively. A threshold of 0.143 was used. **h** Localization precision histogram of the image shown in **b**. The mean localization precision was 14.9 ± 6.9 nm. **i** Dot plot of the absolute values of drift from **f**. The mean drift was 9.2 ± 5.4 nm along the x-axis and 4.1 ± 3.3 nm along the y-axis. The dots represent the drift data points while the central bar represents the mean drift. The upper and lower bars denote one standard deviation.

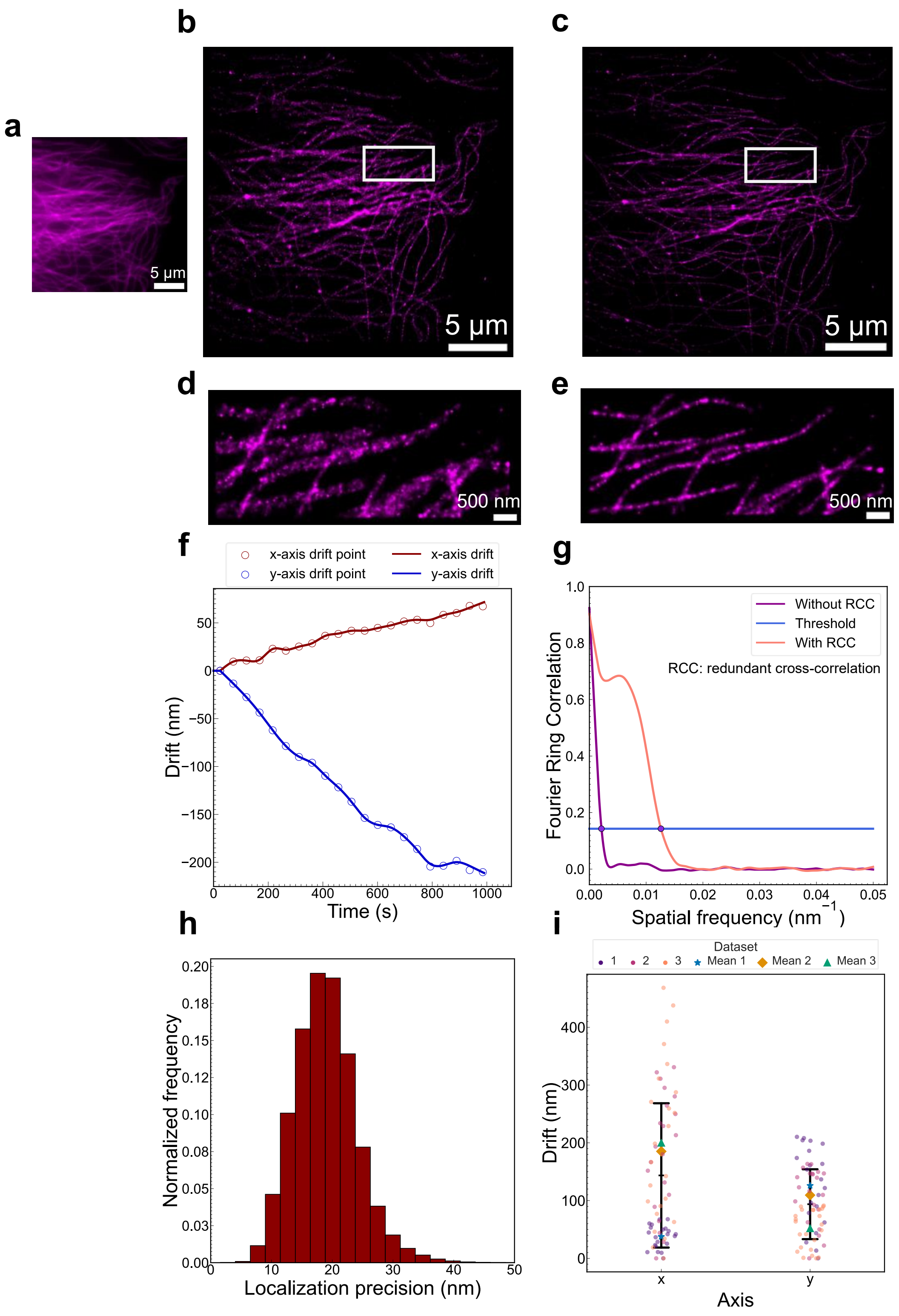

Figure S18. STORM imaging of α-Tubulin-AF647 in the microtubule network in fixed HeLa cells using an off-the-shelf microscope. **a** Widefield image. **b** STORM image without drift correction and **c** drift-corrected STORM image using redundant cross-correlation (RCC). **d** and **e** Magnified view of the boxed area in **b** and **c**. **f** Drift trajectory of the image series from STORM over about 15 minutes (33000 frames). The trajectory was calculated using RCC with each circle representing the drift of one bin, i.e. the drift of one image subset relative to the first subset. A total of 21 bins were used. **g** Fourier ring correlation (FRC) resolution evaluation of the STORM images with (**c**) and without

(**b**) drift correction, demonstrating 312.5 nm and 50.9 nm resolution before and after drift correction, respectively. A threshold of 0.143 was used. **h** Localization precision histogram of the image shown in **b**. The mean localization precision was 19.0 ± 5.2 nm. **i** Dot plot of the mean drifts from three experimental iterations. The dots represent the absolute values of drift data points and are colored according to their dataset. The central black bar represents the mean drift of all three iterations while the upper and lower bars represent one standard deviation. The mean drift of each dataset is represented by a different shape. The mean drift of the first dataset shown above was 37.5 ± 19.4 nm along the x-axis and 125.8 ± 67.1 nm along the y-axis. The mean drift from all iterations was 143.6 ± 124.0 nm along the x-axis and 93.7 ± 60.0 nm along the y-axis.

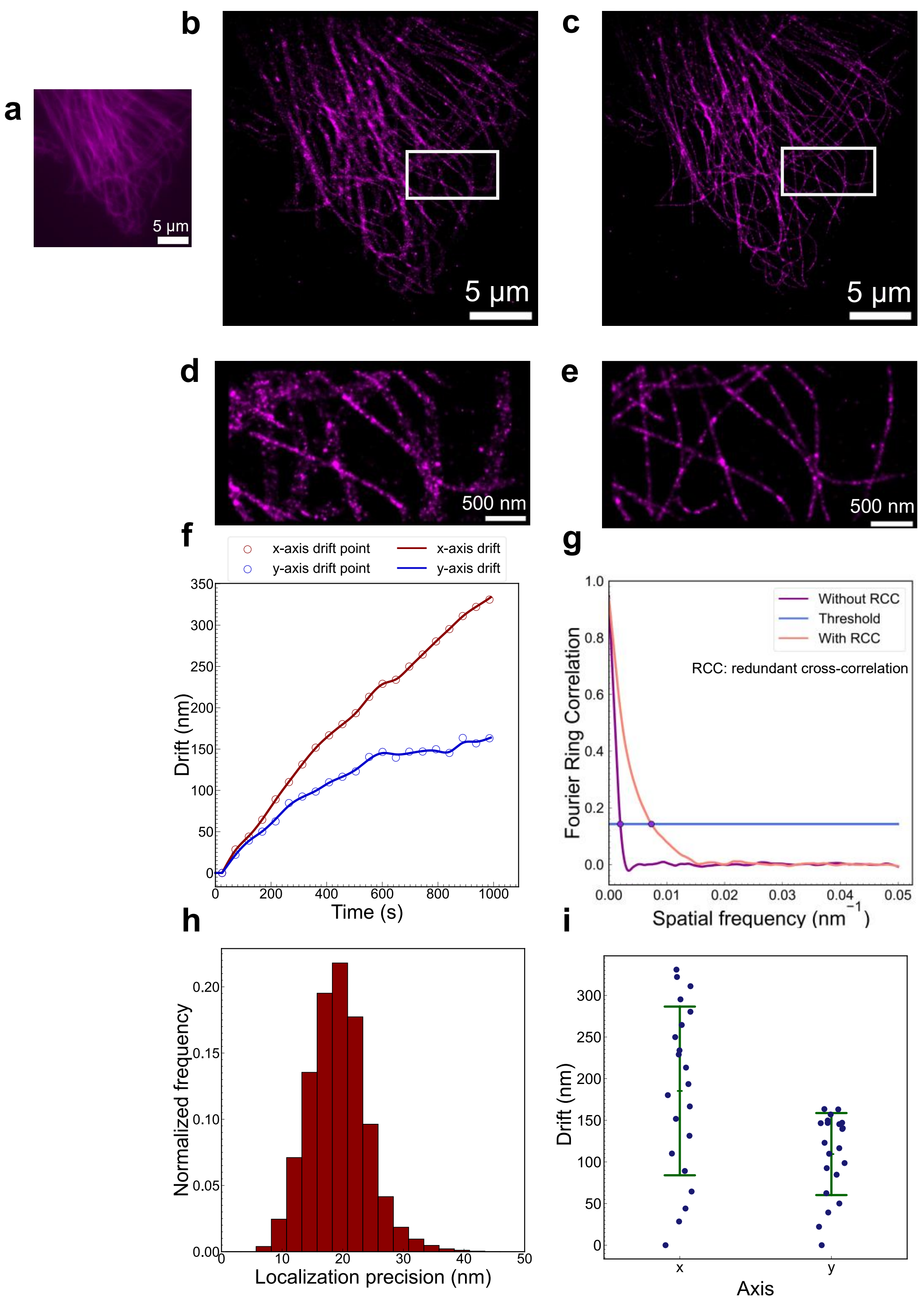

Figure S19. The second iteration of STORM imaging of α-Tubulin-AF647 in the microtubule network in fixed HeLa cells using an off-the-shelf microscope. **a** Widefield image. **b** STORM image without drift correction and **c** drift-corrected STORM image using redundant cross-correlation (RCC). **d** and **e** Magnified view of the boxed area in **b** and **c**. **f** Drift trajectory of the image series from STORM over about 15 minutes (33000 frames). The trajectory was calculated using RCC with each circle representing the drift of one bin, i.e. the drift of one image subset relative to the first subset. A total of 21 bins were used. **g** Fourier ring correlation (FRC) resolution evaluation of the STORM images with (**c**) and without (**b**) drift correction, demonstrating 511.7 nm and 136.5 nm resolution before and after drift correction, respectively. A threshold of 0.143 was used. **h** Localization precision histogram of the image shown

in **b**. The mean localization precision was 19.2 ± 4.8 nm. **i** Dot plot of the absolute values of drift from **f**. The mean drift was 185.2 ± 98.9 nm along the x-axis and 109.4 ± 48.1 nm along the y-axis. The dots represent the drift data points while the central bar represents the mean drift. The upper and lower bars denote one standard deviation.

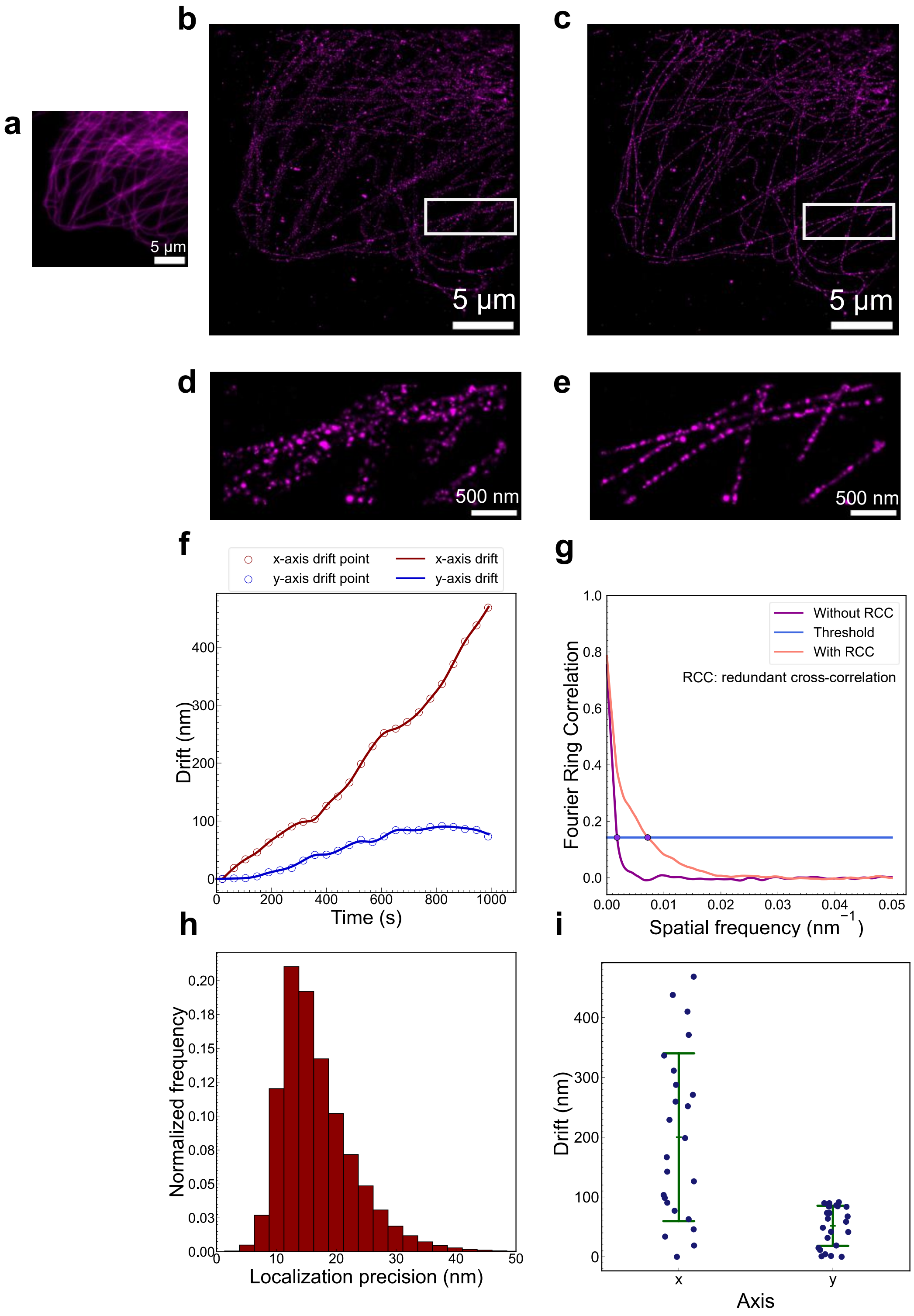

Figure S20. The third iteration of STORM imaging of α-Tubulin-AF647 in the microtubule network in fixed HeLa cells using an off-the-shelf microscope. **a** Widefield image. **b** STORM image without drift correction and **c** drift-corrected

STORM image using redundant cross-correlation (RCC). Scale bar: 5 µm. **d** and **e** Magnified view of the boxed area in **b** and **c**. Scale bar: 0.5 µm. **f** Drift trajectory of the image series from STORM over about 15 minutes (33000 frames). The trajectory was calculated using RCC with each circle representing the drift of one bin, i.e. the drift of one image subset relative to the first subset. A total of 24 bins were used. **g** Fourier ring correlation (FRC) resolution evaluation of the STORM images with (**c**) and without (**b**) drift correction, demonstrating 560.8 nm and 139.2 nm resolution before and after drift correction, respectively. A threshold of 0.143 was used. **h** Localization precision histogram of the image shown in **b**. The mean localization precision was 15.8 ± 6.1 nm. **i** Dot plot of the absolute values of drift from **f**. The mean drift was 199.9 ± 137.3 nm along the x-axis and 51.9 ± 32.8 nm along the y-axis. The dots represent the drift data points while the central bar represents the mean drift. The upper and lower bars denote one standard deviation.

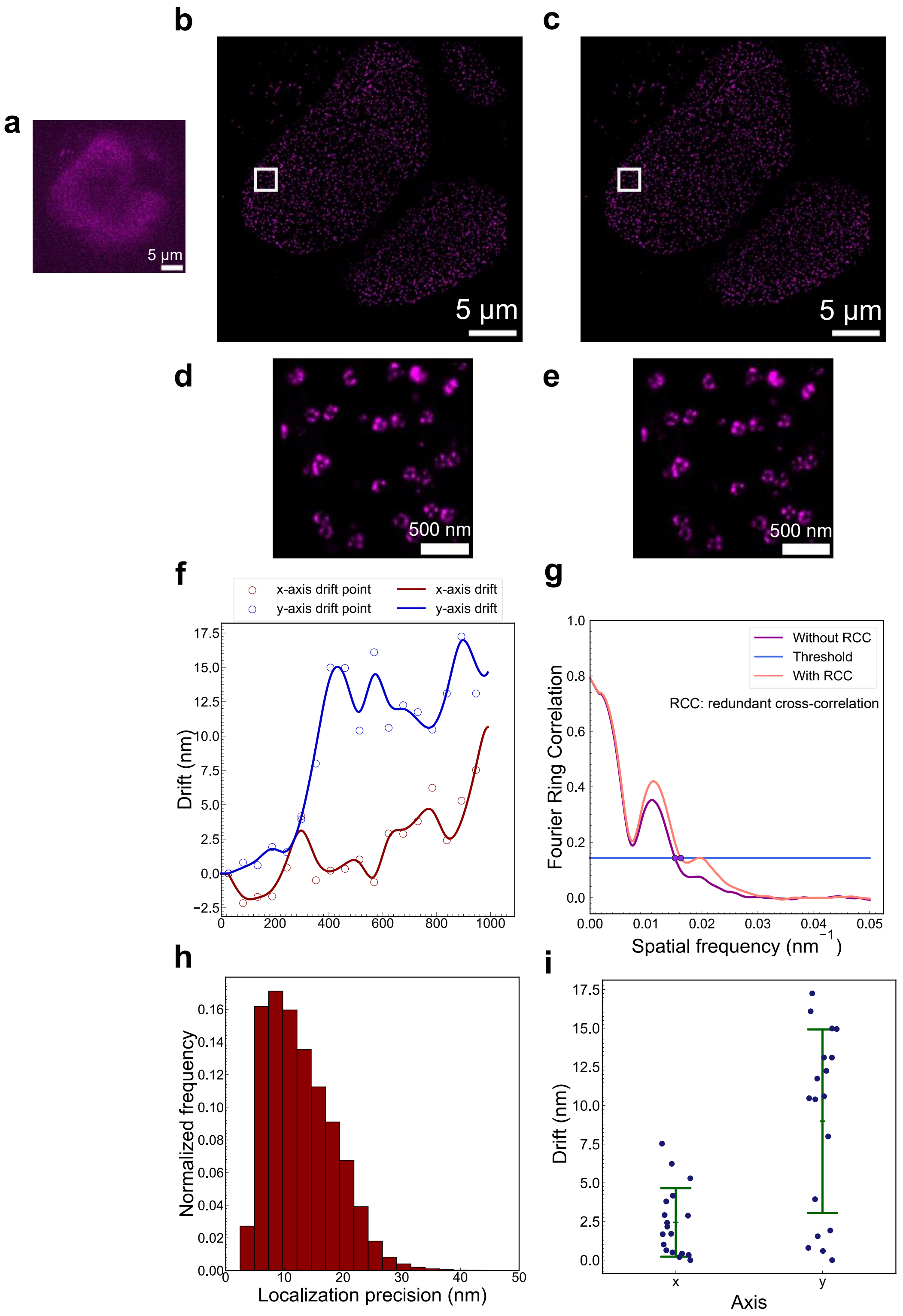

Figure S21. The second iteration of STORM imaging of Nup96-SNAP-AF647 in fixed U2OS cells using the ROCS-STORM system. **a** Widefield image. **b** STORM image without drift correction and **c** drift-corrected STORM image using redundant cross-correlation (RCC). Scale bar: 5 μm. **d** and **e** Magnified view of the boxed area in **b** and **c**. Scale bar: 0.5 μm. **f** Drift trajectory of the image series from STORM over about 15 minutes (33000 frames). The trajectory was calculated using RCC with each circle representing the drift of one bin, i.e. the drift of one image subset relative to the first subset. A total of 17 bins were used. **g** Fourier ring correlation (FRC) resolution evaluation of the STORM

images with (**c**) and without (**b**) drift correction, demonstrating 65.8 nm and 61.7 nm resolution before and after drift correction, respectively. A threshold of 0.143 was used. **h** Localization precision histogram of the image shown in **b**. The mean localization precision was 12.8 ± 5.8 nm. **i** Dot plot of the absolute values of drift from **f**. The mean drift was 2.4 ± 2.2 nm along the x-axis and 9.0 ± 5.8 nm along the y-axis. The dots represent the drift data points while the central bar represents the mean drift. The upper and lower bars denote one standard deviation.

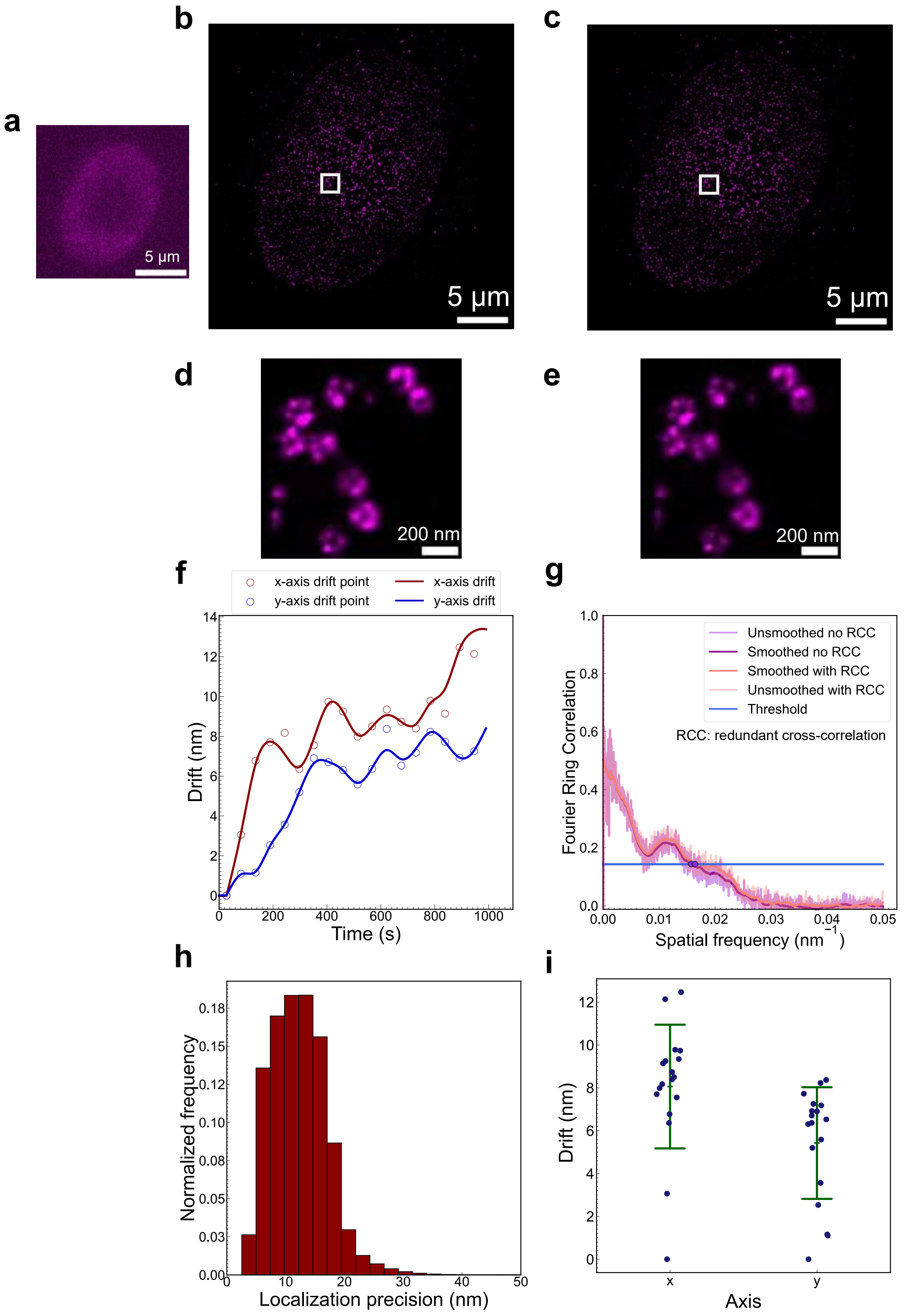

Figure S22. The third iteration of STORM imaging of Nup96-SNAP-AF647 in fixed U2OS cells using the ROCS-STORM system. **a** Widefield image. **b** STORM image without drift correction and **c** drift-corrected STORM image using redundant cross-correlation (RCC). **d** and **e** Magnified view of the boxed area in **b** and **c**. **f** Drift trajectory of the image series from STORM over about 15 minutes (33000 frames). The trajectory was calculated using RCC with each circle representing the drift of one bin, i.e. the drift of one image subset relative to the first subset. A total of 17 bins were used. **g** Fourier ring correlation (FRC) resolution evaluation of the STORM images with (**c**) and without (**b**) drift correction, demonstrating 63.5 nm and 60.9 nm resolution before and after drift correction, respectively. A threshold of 0.143 was used. **h** Localization precision histogram of the image shown in **b**. The mean localization precision was 12.3 ± 4.7 nm. **i** Dot plot of the absolute values of drift from **f**. The mean drift was 8.1 ± 2.8 nm along the x-axis and 5.4 ± 2.5 nm along the y-axis. The dots represent the drift data points while the central bar represents the mean drift. The upper and lower bars denote one standard deviation.

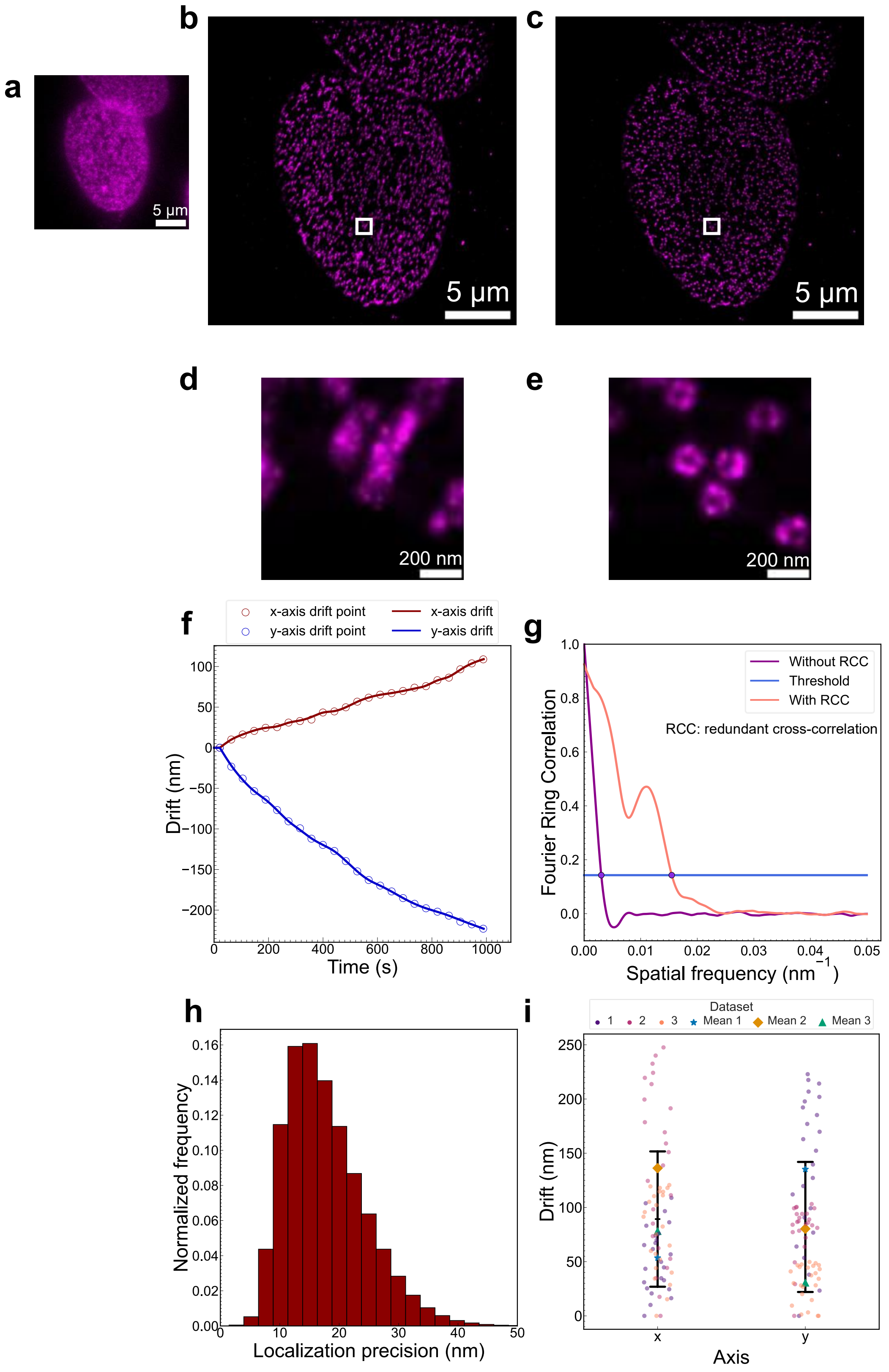

Figure S23. STORM imaging of Nup96-SNAP-AF647 in fixed U2OS cells using an off-the-shelf microscope. **a** Widefield image. **b** STORM image without drift correction and **c** drift-corrected STORM image using redundant cross-correlation (RCC). **d** and **e** Magnified view of the boxed area in **b** and **c**. **f** Drift trajectory of the image series from STORM over about 15 minutes (33000 frames). The trajectory was calculated using RCC with each circle

representing the drift of one bin, i.e. the drift of one image subset relative to the first subset. A total of 24 bins were used. **g** Fourier ring correlation (FRC) resolution evaluation of the STORM images with (**c**) and without (**b**) drift correction, demonstrating 330.2 nm and 64.6 nm resolution before and after drift correction, respectively. A threshold of 0.143 was used. **h** Localization precision histogram of the image shown in **b**. The mean localization precision was 17.7 ± 6.6 nm. **i** Dot plot of the mean drifts from three experimental iterations. The dots represent the absolute values of drift data points and are colored according to their dataset. The central black bar represents the mean drift of all three iterations while the upper and lower bars represent one standard deviation. The mean drift of each dataset is represented by a different shape. The mean drift of the first dataset shown above was 53.4 ± 29.8 nm along the x-axis and 135.2 ± 65.7 nm along the y-axis. The mean drift from all iterations was 89.3 ± 62.0 nm along the x-axis and 82.0 ± 59.6 nm along the y-axis.

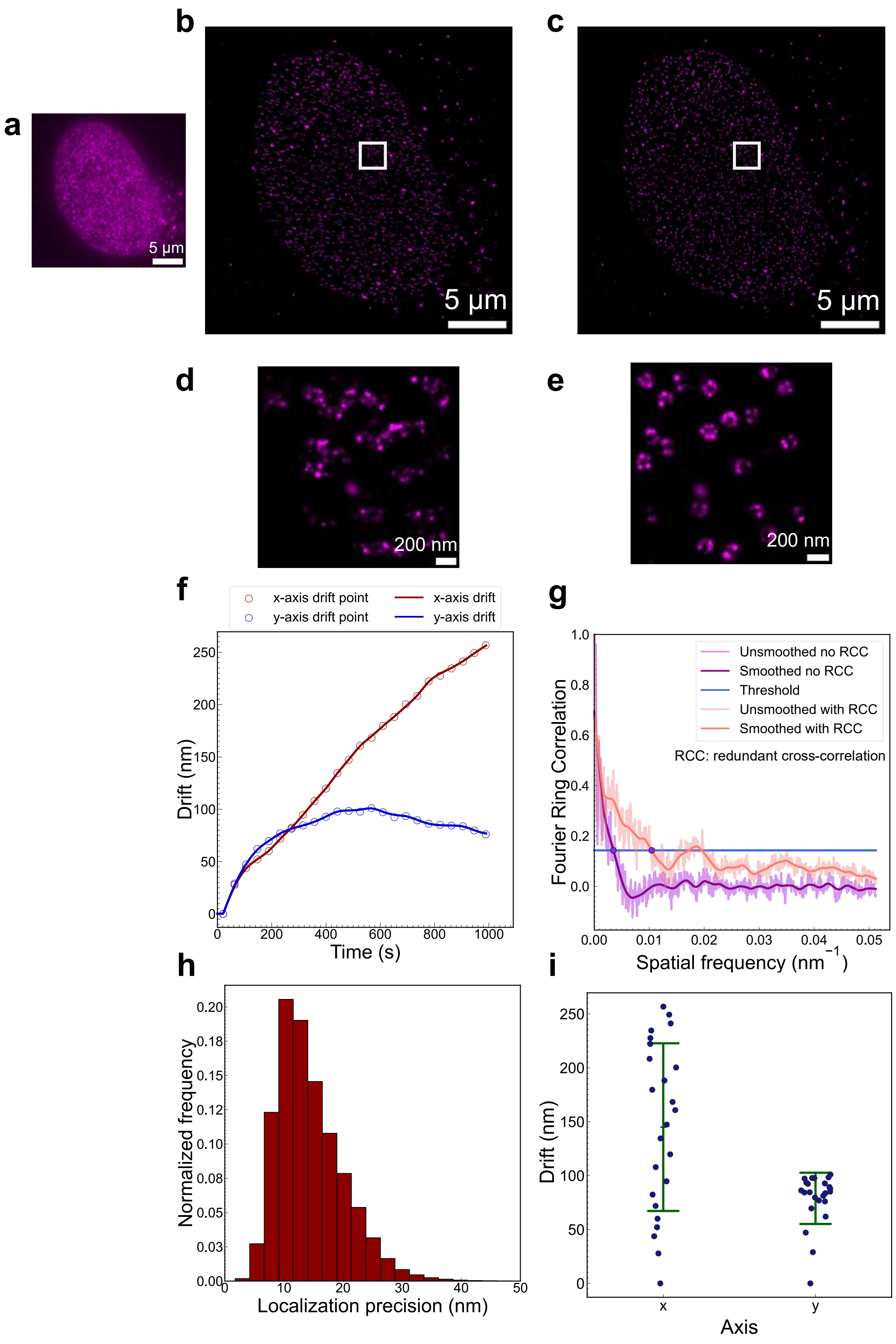

Figure S24. The second iteration of STORM imaging of Nup96-SNAP-AF647 in fixed U2OS cells using an off-the-shelf microscope. **a** Widefield image. **b** STORM image without drift correction and **c** drift-corrected STORM image using redundant cross-correlation (RCC). **d** and **e** Magnified view of the boxed area in **b** and **c**. **f** Drift trajectory of the image series from STORM over about 15 minutes (33000 frames). The trajectory was calculated using RCC with each circle representing the drift of one bin, i.e. the drift of one image subset relative to the first subset. A total of 24 bins were used. **g** Fourier ring correlation (FRC) resolution evaluation of the STORM images with (**c**) and without

(**b**) drift correction, demonstrating 288.1 nm and 95.7 nm resolution before and after drift correction, respectively. A threshold of 0.143 was used. **h** Localization precision histogram of the image shown in **b**. The mean localization precision was 14.5 ± 5.6 nm. **i** Dot plot of the absolute values of drift from **f**. The mean drift was 145.0 ± 76.1 nm along the x-axis and 78.9 ± 23.3 nm along the y-axis. The dots represent the drift data points while the central bar represents the mean drift. The upper and lower bars denote one standard deviation.

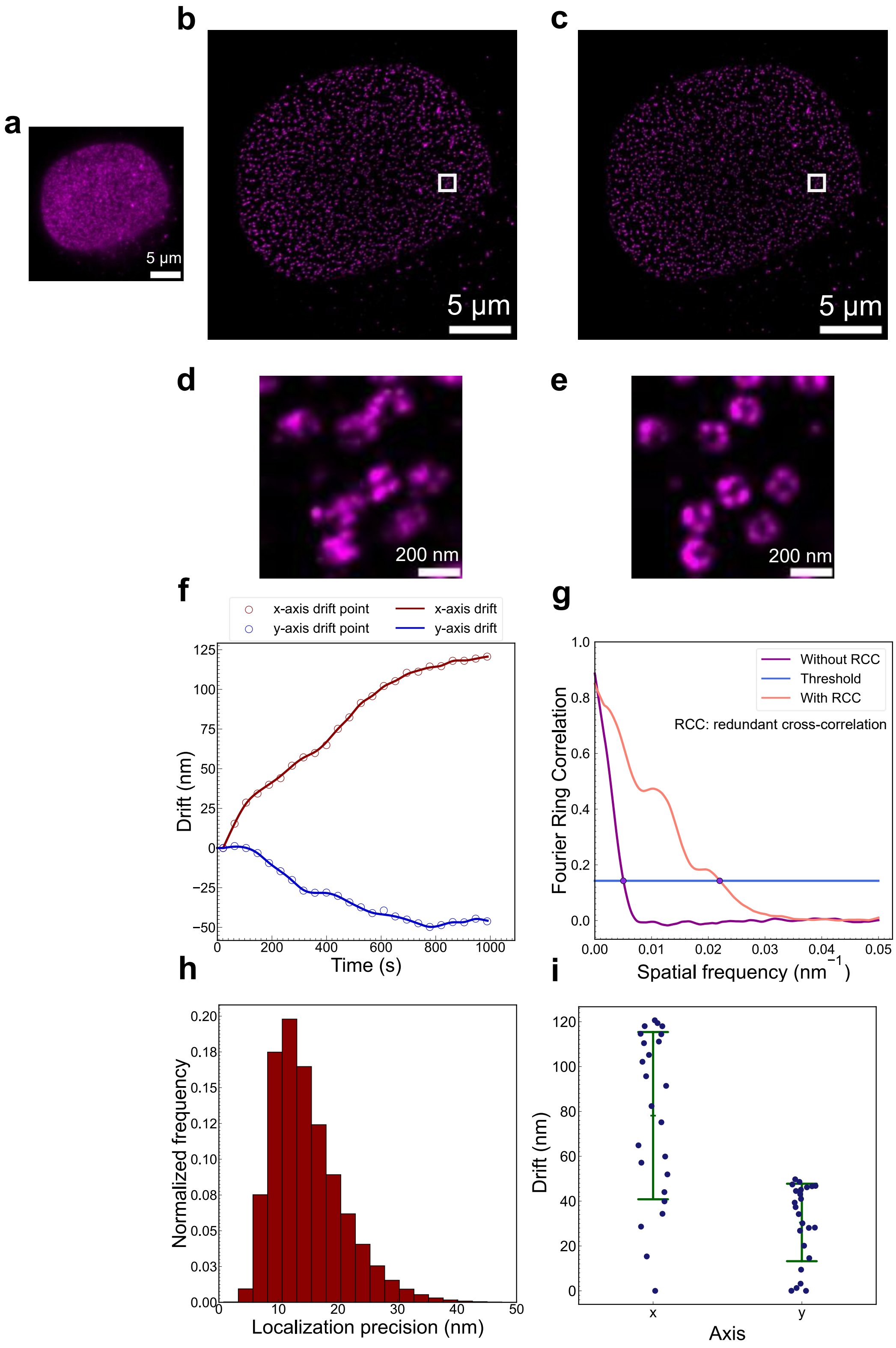

Figure S25. The third iteration of STORM imaging of Nup96-SNAP-AF647 in fixed U2OS cells using an off-the-shelf microscope. **a** Widefield image. **b** STORM image without drift correction and **c** drift-corrected STORM image using redundant cross-correlation (RCC). **d** and **e** Magnified view of the boxed area in **b** and **c**. **f** Drift trajectory of the image series from STORM over about 15 minutes (33000 frames). The trajectory was calculated using RCC with each circle representing the drift of one bin, i.e. the drift of one image subset relative to the first subset. A total of 24 bins were used. **g** Fourier ring correlation (FRC) resolution evaluation of the STORM images with (**c**) and without (**b**) drift correction, demonstrating 198.7 nm and 45.4 nm resolution before and after drift correction, respectively. A threshold of 0.143 was used. **h** Localization precision histogram of the image shown in **b**. The mean localization precision was 14.9 ± 6.0 nm. **i** Dot plot of the absolute values of drift from **f**. The mean drift was 78.1 ± 36.5 nm along the x-axis and 30.5 ± 17.0 nm along the y-axis. The dots represent the drift data points while the central bar represents the mean drift. The upper and lower bars denote one standard deviation.

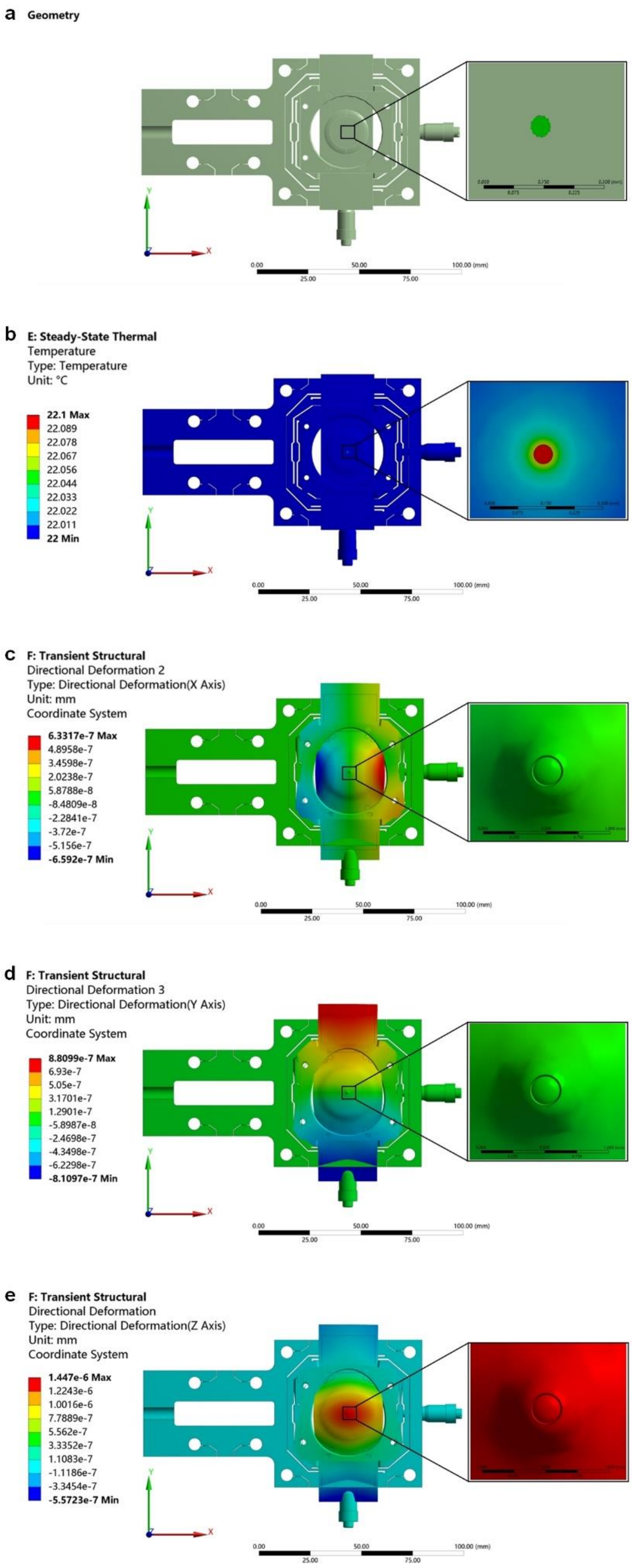
a Geometry
0.00
25.00
50.00
75.00
100.00 (mm)
b E: Steady-State Thermal
Temperature
Type: Temperature
Unit: °C
22.1 Max
22.089
22.078
22.067
22.056
22.044
22.033
22.022
22.011
22 Min
c F: Transient Structural
Directional Deformation 2
Type: Directional Deformation(X Axis)
Unit: mm
Coordinate System
6.3317e-7 Max
4.8958e-7
3.4598e-7
2.0238e-7
5.8788e-8
-8.4809e-8
-2.2841e-7
-3.72e-7
-5.156e-7
-6.592e-7 Min
d F: Transient Structural
Directional Deformation 3
Type: Directional Deformation(Y Axis)
Unit: mm
Coordinate System
8.8099e-7 Max
6.93e-7
5.05e-7
3.1701e-7
1.2901e-7
-5.8987e-8
-2.4698e-7
-4.3498e-7
-6.2298e-7
-8.1097e-7 Min
e F: Transient Structural
Directional Deformation
Type: Directional Deformation(Z Axis)
Unit: mm
Coordinate System
1.447e-6 Max
1.2243e-6
1.0016e-6
7.7889e-7
5.562e-7
3.3352e-7
1.1083e-7
-1.1186e-7
-3.3454e-7
-5.5723e-7 Min

Figure S26. Thermodynamic simulation of the sample stage unit in the ROCS-STORM system. **a** Illustration of the sample stage unit, which includes a sample stage, a coverslide, and a sample holder. The enlarged view of the central region of the coverslide highlights the ϕ 50 µm field-of-view (FOV) in green. This central region is consistently shown in all subsequent figures. **b** The temperature field distribution when the FOV temperature increases by 0.1°C due to laser radiation, with the ambient temperature maintained at 22°C. **c**-**e** Thermal expansion-induced directional deformation along the x, y, and z axes under the conditions outlined in **b**. The results showed maximum thermal expansions of 6.6 nm in the lateral direction and 14 nm in the axial direction on a coverslip, corresponding to a 1 °C temperature increase induced by laser radiation.

Table S1. Parts list of the bench-top ROCS microscope

**Part**	**Mfr**	**Qty**	**Part No.**	**Specifications**	**Unit Price （USD）**	**Notes**
Cage rod	Raycage	12	C08 02 00	L = 200 mm	16.82	
Cage rod		2	C08 05 00	L = 500 mm	44.86	
Cage rod		4	C08 04 00	L = 400 mm	36.45	
Cage rod		4	C08 02 50	L = 250 mm	21.03	
Cage rod		4	C08 01 00	L = 100 mm	8.41	
Cage rod		2	C08 06 00	L= 600 mm	58.88	
No hole holding brake fixed support seat		3	C01 04 11	ØD/MD=Ø6	33.64	
Single-hole brake sliding frame		7	C01 06 11	D=30mm	30.84	
Single hole holding brake fixed support seat		13	C01 01 11	D =30mm	37.15	
Two-hole conversion brake fixed support base		1	C04 01 11	D1=30mm; D2=60mm	64.48	
Card loader		5	C12 20 11	D=25/25.4mm 1mm-6mm	25.23	
Right angle turning pitch angle adjusting		3	C05 01 01	D=25.4mm，1mm-7mm	210.27	

frame						
Sample stage with magnetic holder		1	N/A		490	Custom-made
Multi-function adapter		5	C13 04 21	D=25mm	53.97	
25mm outside diameter column		3	C09 03 00	L= 300 mm	70.09	
Optical plate height manual adjuster		1	C16 03 01	Range: 85 mm	224.26	
Optical plate		1	C15 15 30	150 x 300 mm	98.13	
Holding brake type space right angle turning joint adjusting frame		1	C05 12 14	mirror size:25.4 mm - 36 mm，T＜3 mm	448.52	
Space right angle turning cube		1	C01 08 11	D=35mm	112.13	
XY Translation Mounts for 30 mm Cage Systems	Thorlabs	3	CXY1A		215.57	
Z-axis translation mount		1	SM1ZA	30 mm Cage Compatible	235.61	
30 mm to 60 mm Cage Plate Adapter		1	LCP4S		46.98	

Vertical Cage System Mounting Plate		1	CPVMP		54.67	Blocking the beam for laser safety
25 mm Square Construction Rails		8	XE25L500/M		31.40	
Black posterboard		5	TB5		68.48	
Ø1" achromatic doublets		1	AC254-030-A	f = 30 mm	102.96	
Ø1" achromatic doublets		1	AC254-050-A	f = 50 mm	95.34	
Ø1" achromatic doublets		1	AC254-060-A	f = 60 mm	95.34	
Ø1" achromatic doublets		1	AC254-250-A	f = 250 mm	95.34	
Tube lenses		1	TTL180-A	f = 180 mm	882.40	
Multi-mode fiber		1	M72L01	Ø200 µm, 0.39 NA, Low OH, FC/PC-FC/PC Fiber Patch Cable, 1 m Long	105.83	
Objective	Leica	1	N/A	20x, 0.25*NA*	N/A	Obsolete
Objective	Olympus	1	N1215900	20x, 0.4*NA*	489	
Objective	Olympus	1	N2709400	60x, 1.42*NA*	7,995	Discontinued
PIFOC focus scanners	Physik Instrumente	1	P-721		7,150	Optional
sCMOS camera	Teledyne Photometrics	1	01-Prime-BSI-R-M-16-C		13,231	
Laser combiner	Toptica	1	iChrome-CLE50		41,901	
