# Supplementary material for: Reinforced optical cage systems enable drift-free single-molecule localization microscopy
Source: Commun Eng. 2025 Dec 15;5:13. doi: 10.1038/s44172-025-00566-4 (PMC12820167; doi:10.1038/s44172-025-00566-4)
Supplement: Supplementary file 3 — Description of Additional Supplementary Files [file 44172_2025_566_MOESM3_ESM.pdf]

Description of Additional Supplementary Files:

File: Supplementary Movie 1

Description: Animation of 3D rendering of the ROCS microscope design.
