# Supplementary material for: Reinforced optical cage systems enable drift-free single-molecule localization microscopy
Source: Commun Eng. 2025 Dec 15;5:13. doi: 10.1038/s44172-025-00566-4 (PMC12820167; doi:10.1038/s44172-025-00566-4)
Supplement: Supplementary file 5 — Reporting Summary [file 44172_2025_566_MOESM5_ESM.pdf]

nature portfolio

Corresponding author(s): Lin Wang

Last updated by author(s): Nov 11, 2025

Reporting Summary

Nature Portfolio wishes to improve the reproducibility of the work that we publish. This form provides structure for consistency and transparency in reporting. For further information on Nature Portfolio policies, see our Editorial Policies and the Editorial Policy Checklist.

Statistics

For all statistical analyses, confirm that the following items are present in the figure legend, table legend, main text, or Methods section.

n/a	Confirmed	
☐	☒	The exact sample size (*n*) for each experimental group/condition, given as a discrete number and unit of measurement
☐	☒	A statement on whether measurements were taken from distinct samples or whether the same sample was measured repeatedly
☐	☒	The statistical test(s) used AND whether they are one- or two-sided<br>*Only common tests should be described solely by name; describe more complex techniques in the Methods section.*
☒	☐	A description of all covariates tested
☒	☐	A description of any assumptions or corrections, such as tests of normality and adjustment for multiple comparisons
☐	☒	A full description of the statistical parameters including central tendency (e.g. means) or other basic estimates (e.g. regression coefficient) AND variation (e.g. standard deviation) or associated estimates of uncertainty (e.g. confidence intervals)
☒	☐	For null hypothesis testing, the test statistic (e.g. F, t, r) with confidence intervals, effect sizes, degrees of freedom and P value noted<br>*Give P values as exact values whenever suitable.*
☒	☐	For Bayesian analysis, information on the choice of priors and Markov chain Monte Carlo settings
☒	☐	For hierarchical and complex designs, identification of the appropriate level for tests and full reporting of outcomes
☒	☐	Estimates of effect sizes (e.g. Cohen's d, Pearson's r), indicating how they were calculated

Our web collection on statistics for biologists contains articles on many of the points above.

Software and code

Policy information about availability of computer code

Data collection	3D mechanical design of the ROCS microscope was implemented using commercially available Autodesk Inventor software from Autodesk, Inc. Vibration model finite element analysis was implemented using commercially available ANSYS software from ANSYS, Inc. Image data from the ROCS microscope was collected using open source software Micro-Manager 2.0. Image data from ZEISS AxioObserver Z1 microscope and Elyra PS.1 microscope was collected using commercially available software ZEN 2.3 black edition from Carl Zeiss.
Data analysis	STORM data analysis was implemented using the ThunderSTORM plugin (version 1.3) in open source software ImageJ (version 1.53t). Drift from STORM was quantified using redundant cross-correlation in open source MATLAB-based Super-resolution Microscopy Analysis Platform (SMAP) software (v201217). All drift trajectories were plotted using custom scripts written in Python 3.10 with the NumPy (version 2.1.1), Matplotlib (version 3.9.2), Pandas (version 2.2.2), and Seaborn (0.13.2) packages. FRC curves were plotted using custom scripts written in Python 3.10 with the NumPy (version 2.1.1) and Matplotlib (version 3.9.2) packages.

For manuscripts utilizing custom algorithms or software that are central to the research but not yet described in published literature, software must be made available to editors and reviewers. We strongly encourage code deposition in a community repository (e.g. GitHub). See the Nature Portfolio guidelines for submitting code & software for further information.

Data

Policy information about availability of data

All manuscripts must include a data availability statement. This statement should provide the following information, where applicable:

- Accession codes, unique identifiers, or web links for publicly available datasets
- A description of any restrictions on data availability
- For clinical datasets or third party data, please ensure that the statement adheres to our policy

The datasets generated and analyzed in this study are available in the STFC eData repository, [https://doi.org/10.5286/edata/942], with additional datasets in the Zenodo repository, [https://doi.org/10.5281/zenodo.15407010].

Research involving human participants, their data, or biological material

Policy information about studies with human participants or human data. See also policy information about sex, gender (identity/presentation), and sexual orientation and race, ethnicity and racism.

Reporting on sex and gender	Not applicable.
Reporting on race, ethnicity, or other socially relevant groupings	Not applicable.
Population characteristics	Not applicable.
Recruitment	Not applicable.
Ethics oversight	Not applicable.

Note that full information on the approval of the study protocol must also be provided in the manuscript.

Field-specific reporting

Please select the one below that is the best fit for your research. If you are not sure, read the appropriate sections before making your selection.

☒ Life sciences ☐ Behavioural & social sciences ☐ Ecological, evolutionary & environmental sciences

For a reference copy of the document with all sections, see nature.com/documents/nr-reporting-summary-flat.pdf

Life sciences study design

All studies must disclose on these points even when the disclosure is negative.

Sample size	No formal sample size calculation was performed prior to the study. Sample sizes (n = 3 cells) were chosen based on practical considerations including STORM imaging time constraints, cell availability and standard practice in the field. This sample size provides adequate representation of the biological variability in STORM imaging.
Data exclusions	No data was excluded.
Replication	Reproducibility was assessed by performing STORM experiment on 3 independent occasions of biologically independent samples. The key finding of negligible drift in the STORM imaging using the ROCS microscope was consistently observed across all replicates.
Randomization	Three cells were randomly selected from the available cell population for imaging analysis to assess microscope drift across different instruments. Random selection was employed to minimize selection bias and ensure that drift measurements were representative of typical imaging conditions rather than influenced by specific cell characteristics. Covariate control was not applicable since microscope drift is a technical parameter of the imaging system itself, independent of individual cell properties.
Blinding	Blinding was not applicable to this technical validation study as investigators needed to know which microscope was being used during data collection to properly operate the equipment and record drift measurements. Since the primary outcome, i.e. microscope drift, is an objective, quantitative measurement obtained through automated image analysis software, investigator knowledge of the microscope identity does not introduce subjective bias into the data collection or analysis process.

Reporting for specific materials, systems and methods

We require information from authors about some types of materials, experimental systems and methods used in many studies. Here, indicate whether each material, system or method listed is relevant to your study. If you are not sure if a list item applies to your research, read the appropriate section before selecting a response.

Materials & experimental systems

n/a	Involved in the study
☐	☒ Antibodies
☐	☒ Eukaryotic cell lines
☒	☐ Palaeontology and archaeology
☒	☐ Animals and other organisms
☒	☐ Clinical data
☒	☐ Dual use research of concern
☒	☐ Plants

Methods

n/a	Involved in the study
☒	☐ ChIP-seq
☒	☐ Flow cytometry
☒	☐ MRI-based neuroimaging

Antibodies

Antibodies used

Anti-α-tubulin primary antibodies (Catalog No. T5168, monoclonal mouse antibody, Sigma-Aldrich); donkey anti-mouse IgG (H+L) highly cross-adsorbed secondary antibody, Alexa Fluor plus 647 (Catalog No. A32787, Thermo Fisher Scientific)

Validation

Anti-α-tubulin primary antibodies (Catalog No. T5168, monoclonal mouse antibody, Sigma?Aldrich) are widely used for super?resolution microscopy, including STORM imaging of microtubule networks in fixed HeLa cells, as described by Jimenez, A., Friedl, K. & Leterrier, C. in About samples, giving examples: Optimized Single Molecule Localization Microscopy. Methods 174, 100–114 (2020).

Eukaryotic cell lines

Policy information about cell lines and Sex and Gender in Research

Cell line source(s)

HeLa (ATCC CCL-2) originated from a 1951 cervical tumor biopsy of Henrietta Lacks, an African American woman. U2OS cells endogenously expressing Nup96-SNAP (Catalog No. 300444, Cell Line Service) were derived from a moderately differentiated osteosarcoma of the tibia from a 15-year-old white female patient, established in 1964.

Authentication

Cell lines were not authenticated.

Mycoplasma contamination

Cells were tested and confirmed negative for mycoplasma using standard DAPI stain and/or Lookout Mycoplasm PCR Detection Kit (Sigma, Cat no. MP0035).

Commonly misidentified lines
(See ICLAC register)

The cell lines are not listed in the misidentified cell registry.

Plants

Seed stocks

Report on the source of all seed stocks or other plant material used. If applicable, state the seed stock centre and catalogue number. If plant specimens were collected from the field, describe the collection location, date and sampling procedures.

Novel plant genotypes

Describe the methods by which all novel plant genotypes were produced. This includes those generated by transgenic approaches, gene editing, chemical/radiation-based mutagenesis and hybridization. For transgenic lines, describe the transformation method, the number of independent lines analyzed and the generation upon which experiments were performed. For gene-edited lines, describe the editor used, the endogenous sequence targeted for editing, the targeting guide RNA sequence (if applicable) and how the editor was applied.

Authentication

Describe any authentication procedures for each seed stock used or novel genotype generated. Describe any experiments used to assess the effect of a mutation and, where applicable, how potential secondary effects (e.g. second site T-DNA insertions, mosiacism, off-target gene editing) were examined.
